# Tamoxifen exerts anti-peritoneal fibrosis effects by inhibiting H19-activated *VEGFA* transcription

**DOI:** 10.1186/s12967-023-04470-3

**Published:** 2023-09-11

**Authors:** Tingting Zhao, Zhengyu Sun, Xueli Lai, Hongtao Lu, Lulu Liu, Shuangxi Li, Ji-hang Yuan, Zhiyong Guo

**Affiliations:** 1 Department of Nephrology, First Affiliated Hospital of Naval Medical University, Shanghai Changhai Hospital, Shanghai, 200433 China; 2 Department of Nephrology, Shanghai Jiao Tong University Affiliated Sixth People’s Hospital, Shanghai, 200433 China; 3 Department of Nutrition, Naval Medical University, Shanghai, 200433 China; 4 Department of Medical Genetics, Naval Medical University, Shanghai, 200433 China

**Keywords:** Peritoneal fibrosis, Tamoxifen, Estrogen receptor 1, Long non-coding RNA H19, Vascular endothelial growth factor A

## Abstract

**Background:**

Peritoneal dialysis (PD) remains limited due to dialysis failure caused by peritoneal fibrosis. Tamoxifen (TAM), an inhibitor of estrogen receptor 1 (ESR1), has been reported to treat fibrosis, but the underlying mechanism remains unknown. In this study, we sought to explore whether tamoxifen played an anti-fibrotic role by affecting transcription factor ESR1.

**Methods:**

ESR1 expression was detected in the human peritoneum. Mice were daily intraperitoneally injected with 4.25% glucose PD dialysate containing 40 mM methylglyoxal for 2 weeks to establish PD-induced peritoneal fibrosis. Tamoxifen was administrated by daily gavage, at the dose of 10 mg/kg. Chromatin immunoprecipitation (ChIP) and dual‐luciferase reporter assay were performed to validate ESR1 bound *H19* promoter. Gain-of-function and loss-of-function experiments were performed to investigate the biological roles of H19 on the mesothelial-mesenchymal transition (MMT) of human peritoneal mesothelial cells (HPMCs). Intraperitoneal injection of nanomaterial-wrapped 2′-*O*-Me-modified small interfering RNA was applied to suppress H19 in the mouse peritoneum. RNA immunoprecipitation and RNA pull-down assays demonstrated binding between H19 and p300. Exfoliated peritoneal cells were obtained from peritoneal dialysis effluent to analyze the correlations between ESR1 (or H19) and peritoneal solute transfer rate (PSTR).

**Results:**

ESR1 was increased significantly in the peritoneum after long-term exposure to PD dialysate. Tamoxifen treatment ameliorated high glucose-induced MMT of HPMCs, improved ultrafiltration rate, and decreased PSTR of mouse peritoneum. Tamoxifen reduced the H19 level by decreasing the ESR1 transcription of *H19*. Depletion of H19 reversed the pro-fibrotic effect of high glucose while ectopic expression of H19 exacerbated fibrotic pathological changes. Intraperitoneal injection of nanomaterial-wrapped 2′-*O*-Me-modified siRNAs targeting H19 mitigated PD-related fibrosis in mice. RNA immunoprecipitation (RIP) and RNA pull-down results delineated that H19 activated VEGFA expression by binding p300 to the *VEGFA* promoter and inducing histone acetylation of the *VEGFA* promoter. ESR1 and H19 were promising targets to predict peritoneal function.

**Conclusions:**

High glucose-induced MMT of peritoneal mesothelial cells in peritoneal dialysis via activating ESR1. In peritoneal mesothelial cells, ESR1 transcribed the *H19* and H19 binds to transcription cofactor p300 to activate the *VEGFA*. Targeting ESR1/H19/VEGFA pathway provided new hope for patients undergoing peritoneal dialysis.

**Graphic Abstract:**

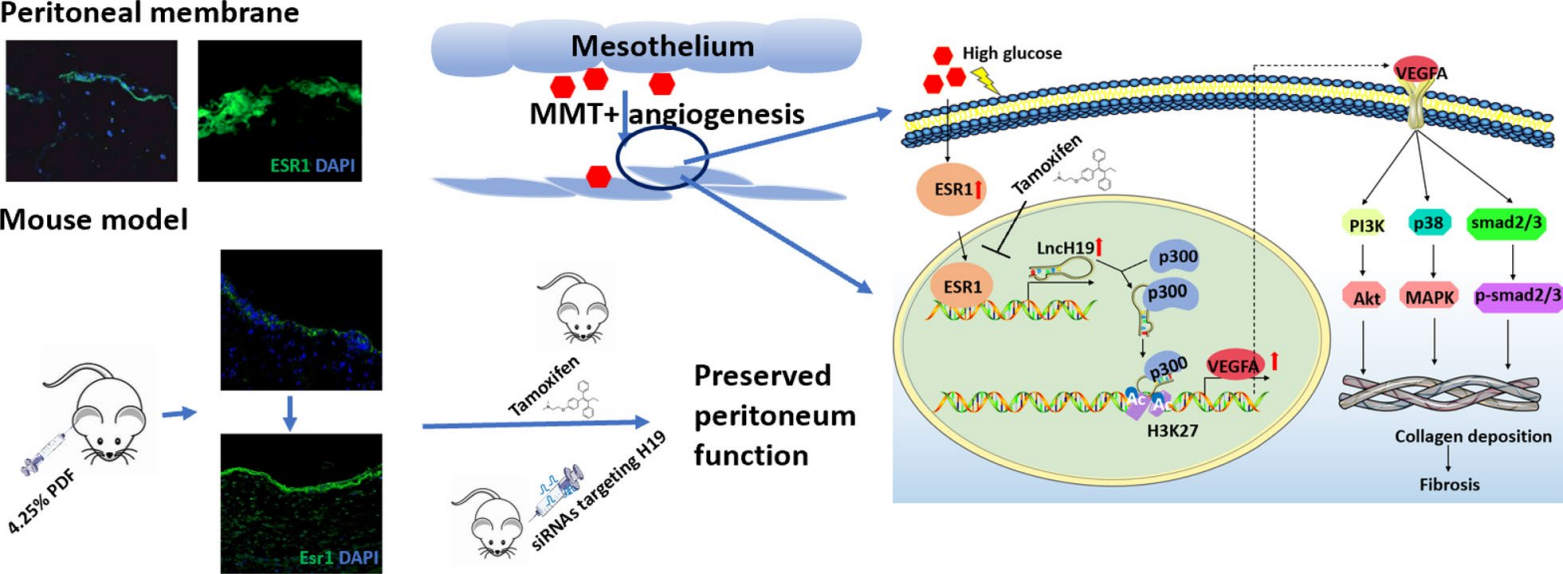

**Supplementary Information:**

The online version contains supplementary material available at 10.1186/s12967-023-04470-3.

## Introduction

Approximately 10% of the global population suffers from chronic kidney disease, with a higher prevalence among elderly people [1]. Peritoneal dialysis (PD) is one of the principal treatments for patients with renal failure due to its simplicity and low costs, providing a similar survival rate compared with hemodialysis [2]. The mainstay of osmotic agents in the hypertonic dialysate is glucose, which provides osmotic pressure in the form of 1.5%, 2.5%, or 4.25% dextrose (glucose monohydrate) [1]. However, long-term exposure to glucose increases the number of peritoneal vessels and the severity of vasculopathy in the peritoneum [3]. As a result, patients with greater peritoneal arteriolar vascularity have poor ultrafiltration. Previous studies have confirmed that the production of vascular endothelial growth factor A (VEGFA), a potent proangiogenic cytokine, is induced by high-glucose peritoneal dialysis fluid (PDF) and secreted by mesothelial cells that have undergone mesothelial-mesenchymal transition (MMT) [4–6]. VEGFA not only binds to receptor tyrosine kinase VEGFR2 to promote differentiation and proliferation of endothelial cells but also mediates TGF-β/smad or other pathways activation of the peritoneal mesothelial cells by autocrine secretion [7, 8]. When VEGFA receptors are triggered, it activates feed-forward amplification loops of fibrosis in peritoneal mesothelial cells. Thus, VEGFA plays a vital role in the deterioration of peritoneal fibrosis by promoting MMT of peritoneal cells and angiogenesis and has become a target for easing PD-related fibrosis [9].

Tamoxifen (TAM) is a selective estrogen receptor modulator (SERM) for treating breast cancer. In parallel, tamoxifen has been validated to achieve excellent efficiency in chronic fibrotic diseases such as fibrosing mediastinitis, idiopathic sclerosing cervicitis, Dupuytren’s palmar fascia, and rapidly growing desmoid tumors [10, 11]. Clark reported dramatic reductions in peritoneal fibrosis and mortality in two patients with retroperitoneal fibrosis who were treated orally with tamoxifen in 1991 [12]. Tamoxifen acts as an agonist or an inhibitor of estrogen receptor 1 (ESR1), depending on ESR1 expression levels, ESR1/ESR2 ratios, and in vivo tamoxifen metabolites [13]. It displays dual biologic responses depending on the tissues exposed. However, the role of tamoxifen in regulating ESR1 remains unclear in peritoneal dialysis-related fibrosis. We thus develop an interest in how tamoxifen protects the peritoneum by modulating ESR1. Considering the classic nuclear/genomic actions of ESR1 as a transcription factor to control gene expression [13], we aimed to investigate whether tamoxifen is involved in the modulation of DNA genomic transcription to mitigate peritoneal fibrosis.

Genomic analyses have revealed that approximately 90% of human genome sequences are transcribed into noncoding RNAs, which have critical regulatory roles in multiple biological processes [14]. Long noncoding RNAs (LncRNAs), which are longer than 200 nucleotides, have been discovered to exert both pro- and anti-fibrotic effects on the multifaceted processes of fibrosis [15]. H19 is one of the few long noncoding RNAs conserved between mice and human [16]. Circulating H19 levels in plasma were identified as a promising biomarker of hypertrophy and fibrosis of the right ventricular failure [17]. H19 released into circulation by exosomal package was positively correlated with the severity of the fibrotic liver injury [18, 19]. However, the function of H19 in peritoneal dialysis-related fibrosis has not been demonstrated. Therefore, our study investigated whether tamoxifen plays an inhibitory role in fibrosis by interfering with ESR1 translocation into the nucleus to transcribe *H19*.

Acetylation of histone lysine residues on nucleosomes was first identified as a reversible modification and has been extensively studied in the regulation of chromatin structure and function [20]. The transcriptional coactivator p300 is a histone acetyltransferase (HAT) that is typically recruited to a transcriptional promoter and regulates gene expression by acetylating chromatin. p300 has been reported to be essential for the transcription of *VEGFA*, which not only forms the p300/HIF-α complex but also acetylates histone H3 at lysine 27 (H3K27ac), a well-defined marker of activating transcription [21, 22]. In response to environmental and/or developmental cues, promoters impart spatiotemporal specificity to gene expression, where LncRNA binds to the HAT domain, increasing H3K27ac at the associated promoter [23].

In this study, we clarified the mechanism of tamoxifen in alleviating high glucose-induced fibrosis by inhibiting ESR1 transcribing *H19* in peritoneal fibrosis. In peritoneal dialysis, high glucose promoted ESR1 transcribed *H19* and H19 bound p300 to activate the *VEGFA* promoter by acetylating histone H3 at lysine 27.

## Methods

Details for the methods are given in the Additional file 1: materials and methods.

### Human peritoneum specimens

Human peritoneum specimens were collected from Shanghai Changhai Hospital. Six normal peritoneal tissues were obtained from patients receiving catheterization for PD. Six long-term PD samples were obtained from patients receiving extubating procedures who suffered peritoneal ultrafiltration failure caused by peritoneal fibrosis and whose PD durations were all over 5 years. Patients’ information was supplied in Additional file 1: Table S1a. All patients were informed and consented to the procedure. The Shanghai Changhai Hospital Ethics Committee approved our study (CHBC2020-096)**.**

### Human peritoneal mesothelial cell collection

Patients on glucose-based PD regimens undergoing continuous ambulatory peritoneal dialysis (CAPD) were recruited and all patients used PDF from Dianeal Baxter company in the center of the Nephrology Department of Shanghai Changhai Hospital. The exclusion criteria were the same as the previous studies, including diagnosis of peritonitis within the past 6 months, active autoimmune diseases, active inflammatory processes, and malignant tumors [24, 25]. Demographic characteristics were recorded in our study: age, gender, PD duration, cause of ESRD, and Peritoneal Equilibrium Test (D/P Cr). Human peritoneal epithelial cells were obtained from peritoneal dialysis effluent with dwell times ranging from 7 to 10 h and cultured as described [26]. This experiment was approved by the Ethics Committee of Shanghai Changhai Hospital. All patients were informed and consented to the procedure. Clinical information statistics were in the Additional file 1: Table S1b.

### Animal study

All animal experiments followed the guidelines and protocols of the Animal Ethics Committee of the Naval Medical University. Methods for PDF-induced peritoneal fibrosis, tamoxifen administration mouse model, and the intraperitoneal delivery of liposomal siRNAs were detailed in Additional file 1: materials and methods.

### Cellular experiment

Human peritoneal mesothelial cells, the MeT-5A cell line, were purchased from ATCC. Mouse primary peritoneal mesothelial cells were obtained by 0.25% trypsin–EDTA digestion of the parietal peritoneum, sorted by flow cytometry.

### Statistics

Data are expressed as means ± SD. Statistical analyses were performed with GraphPad Prism (version 8.0, GraphPad Software, San Diego, CA). The Kolmogorov–Smirnov normality test was performed to test value distribution. A two-tailed Student’s t-test was used for the comparison of the two groups. One-way ANOVA was used followed by Dunnett’s T3. Tukey’s multiple comparisons were tested following Two-way ANOVA. The correlation between the two variables was analyzed by Pearson correlation. Significance was defined as *P* < 0.05.

## Results

### ESR1 was upregulated in the peritoneal mesothelial cells after PDF exposure

**Fig. 1:**
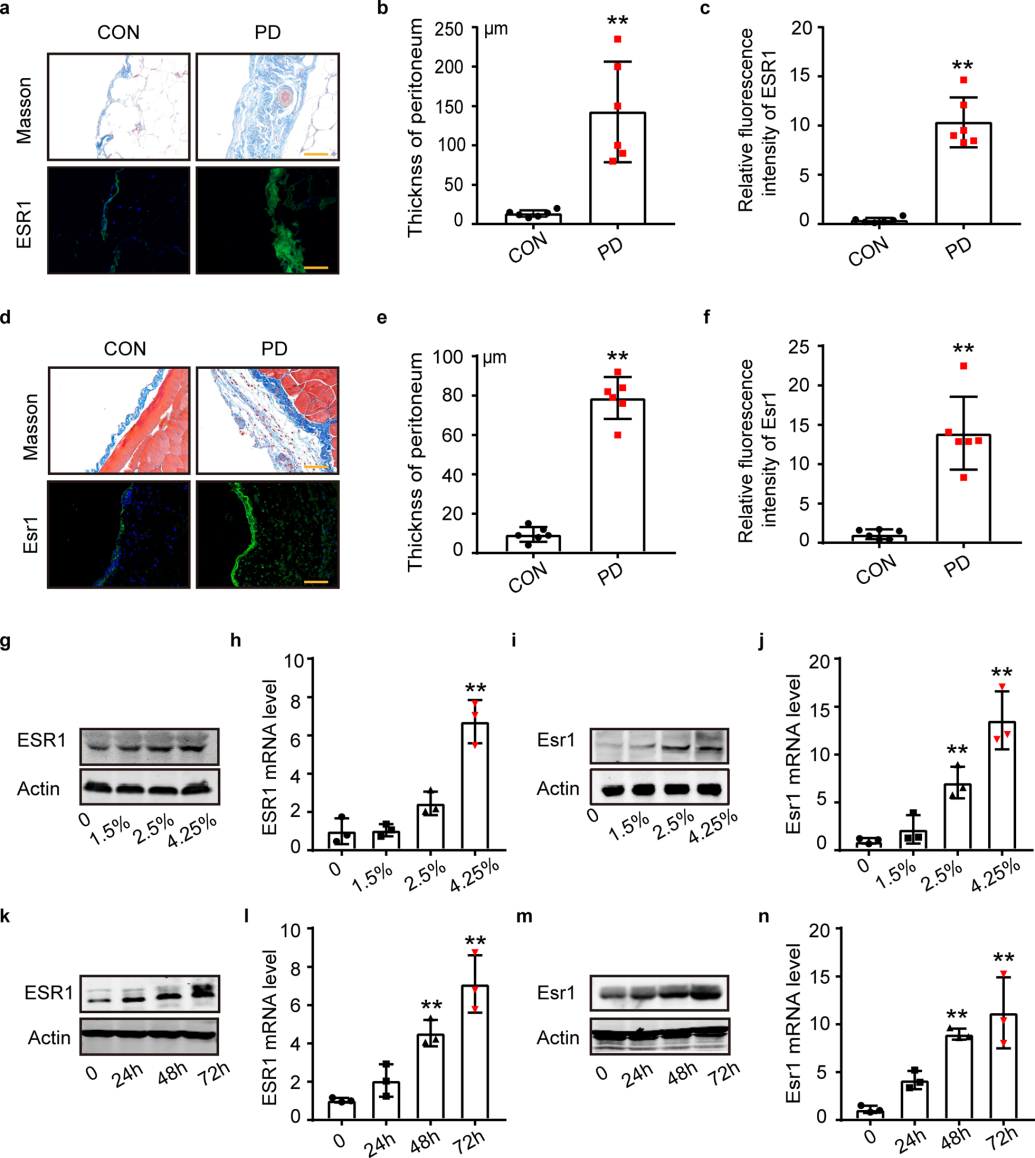
ESR1 was upregulated in the peritoneal mesothelial cells after PDF exposure.** a** Masson staining and immunofluorescence images of human peritoneum. Scale bars = 100 μm. **b** Relative quantification of peritoneal thickness in clinical samples. n = 6 samples. **P < 0.01 vs. CON group. **c** ESR1 fluorescence intensity based on acquired images. n = 6. **P < 0.01 vs. CON group. **d** Masson staining and immunofluorescence images of mouse peritoneum. Mice were intraperitoneally injected with saline in the CON group and 4.25% high-glucose peritoneal dialysate containing 40 mM methylglyoxal in the PD group (0.1 ml/g) for 2 weeks. Scale bars = 100 μm. **e** and **f** Relative quantification of the peritoneal thickness and Esr1 fluorescence intensity in the mouse model by ImageJ. n = 6. **P < 0.01 vs. CON group. **g** and** h** Western blot analysis of ESR1 protein and qRT-PCR analysis of ESR1 mRNA expression in the MeT-5A cell line under different glucose concentrations. Concentrations of 1.5%, 2.5%, and 4.25% are commonly used in clinical peritoneal dialysis solutions. n = 3. **P < 0.01 vs. 0% glucose group. **i** and **j** Western blot analysis of Esr1 protein and qRT-PCR analysis of Esr1 mRNA expression in mouse peritoneal primary cells stimulated with different glucose concentrations. Mouse peritoneal primary cells were obtained by intraperitoneal injection of EDTA-trypsin, digested at 37 ℃ for 15 min and sorted by flow cytometry. n = 3 **P < 0.01 vs. 0% glucose group. **k** and** l** ESR1 protein and mRNA level in Met-5A cells stimulated by 4.25% d-glucose with time extension. n = 3 **P < 0.01 vs. 0% glucose group. **m** and **n** Esr1 protein and mRNA level in mouse peritoneal primary cells stimulated by 4.25% d-glucose with time extension. n = 3 **P < 0.01 vs. 0% glucose group. Values are the mean ± SD. Clinical and mouse values came from a Gaussian distribution by normality test. The pairwise comparison between groups was tested by Dunnett's T3 following One-way ANOVA. *ESR1* oestrogen receptor 1, *PD* peritoneal dialysis, *PDF* peritoneal dialysis fluid

To investigate the peritoneal ESR1 level of patients enduring long-term peritoneal dialysis, we obtained peritoneal biopsy samples from patients with catheterization or extubation of peritoneal dialysis (6 vs*.* 6). Masson staining of human peritoneal specimens revealed a marked increase in peritoneal thickness in patients enduring long-term peritoneal dialysis (Fig. 1a, b). The immunofluorescence (IF) intensity of ESR1 was also significantly enhanced in the human peritoneal layer after PDF exposure (Fig. 1a, c). In the PD murine model, the peritoneal thickness of the PD group increased dramatically, and there was a marked rise in Esr1 (Fig. 1d–f). In the human peritoneal mesothelial cell line, MeT-5A, ESR1 protein and mRNA levels were increased by glucose in a dose-dependent manner (Fig. 1g, h). The same conclusion was drawn from mouse primary peritoneal cells (Fig. 1i, j). Moreover, 4.25% d-glucose promoted ESR1 protein and mRNA levels with time extension in MeT-5A cells (Fig. 1k, l) and mouse primary peritoneal cells (Fig. 1m, n).

### Tamoxifen reversed PD-related peritoneal fibrosis

**Fig. 2:**
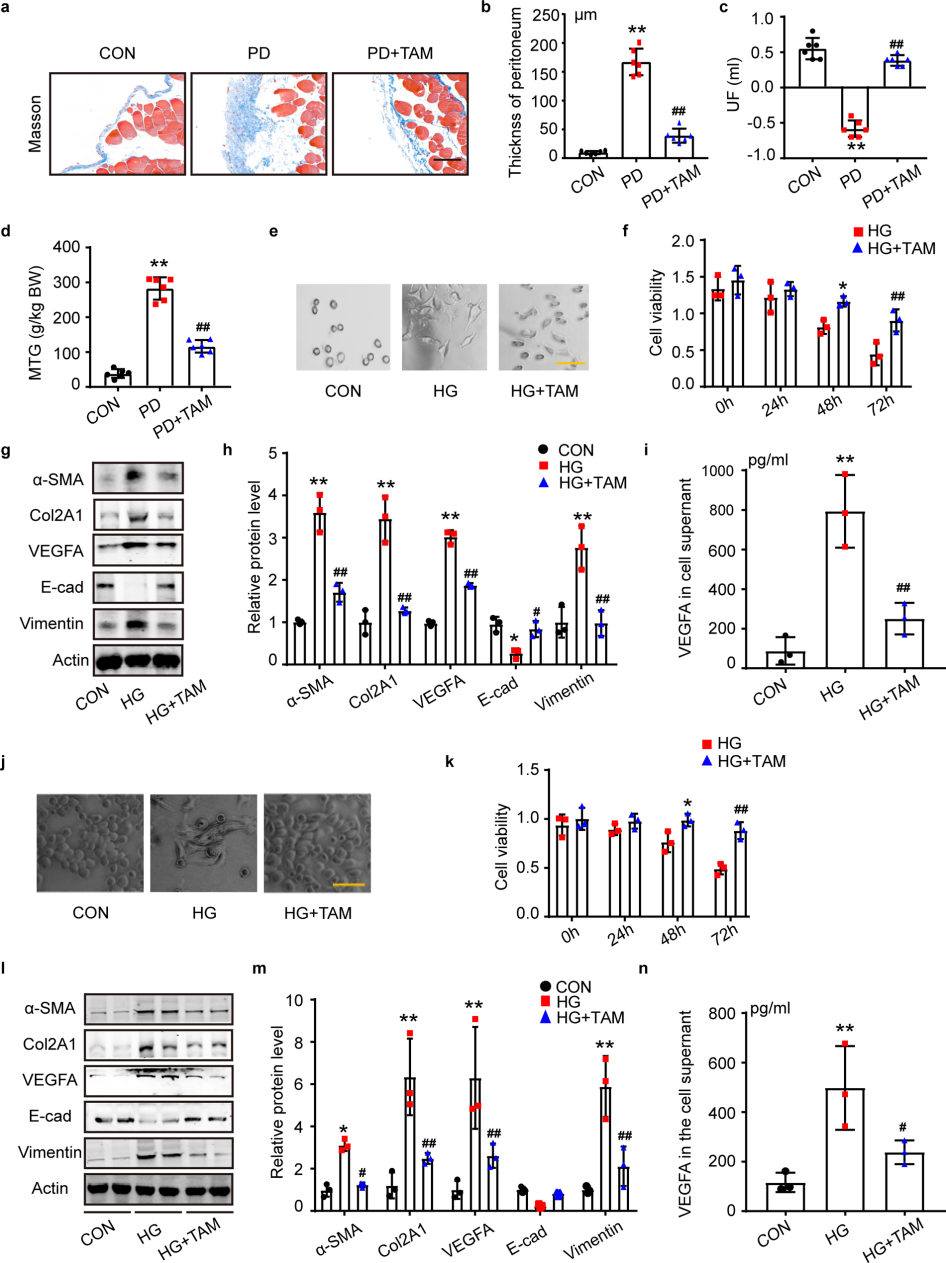
Tamoxifen reversed PD-related peritoneal fibrosis. **a** Masson-stained images showed peritoneal collagen deposition in the mouse model. The CON group received a daily intraperitoneal injection of saline (0.1 ml/g). The PD group received an equal volume of 4.25% daily. Mice in the PD + TAM group received an injection of PDF and tamoxifen citrate treatment, 10 mg/kg, daily, by gavage. n = 6 mice. Scale bars = 100 μm. **b** Relative quantification of peritoneal thickness in the mouse model. n = 6. **P < 0.01 vs. control group, ^**##**^*P* < 0.01 vs. PD group mice. **c** and **d** Peritoneal function test of UF measurements and mass transfer of glucose (MTG). n = 6. **P < 0.01 vs. CON group, ^**##**^*P* < 0.01 vs. PD group. **e** Morphology of mouse peritoneal primary cells. Scale bars = 100 μm. **f** CCK-8 assay showed the viability of mouse peritoneal primary cells under HG stimulation with or without tamoxifen treatment. n = 3. *P < 0.05 vs. 48 h HG group, ^**##**^*P* < 0.01 vs. 72 h HG group. **g** Western blot analysis of EMT and fibrosis markers in mouse peritoneal primary cells. **h** Statistical data analysis of protein Western Blot results. n = 3. *P < 0.05, **P < 0.01 vs. CON group, ^**#**^*P* < 0.05, ^**##**^*P* < 0.01 vs. HG group. **i** VEGFA ELISA of mouse peritoneal primary cells. n = 3. *p < 0.05, **P < 0.01 vs. CON group, ^**#**^*P* < 0.05, ^**##**^*P* < 0.01 vs. HG group. **j** Images of MeT-5A cell morphology captured by microscopy. Scale bars = 100 μm.** k** CCK8 assay showed the viability of MeT-5A cells with or without tamoxifen treatment. n = 3. *P < 0.05, vs. 48 h HG group, ^**##**^*P* < 0.01 vs. 72 h HG group. **l** Western blot analysis of EMT and fibrosis markers in MeT-5A cells. **m** Statistical data analysis of protein Western Blot results. n = 3. *P < 0.05, **P < 0.01 vs. CON group, ^**#**^*P* < 0.05, ^**##**^*P* < 0.01 vs. HG group. **n** VEGFA ELISA of MeT-5A cell supernatant. n = 3. **P < 0.01 vs. CON group, ^**#**^*P* < 0.05 vs. HG group. *TAM* tamoxifen, *HG* 4.25% glucose, *UF* ultrafiltration failure, *MTG* mass transfer of glucose. Cellular studies are performed three times. Values are the mean ± SD. The pairwise comparison between groups was tested by Dunnett's T3 following One-way ANOVA

Masson staining results showed that tamoxifen reversed PDF-induced peritoneal collagen deposition and reduced the thickness of the peritoneum (Fig. 2a, b). In the PD group, we observed a noticeable decline in the ultrafiltration rate (UF) and a significant increase in the mass transfer of glucose (MTG), which were both reversed by TAM (Fig. 2c, d). Normal mouse peritoneal primary cells are similar in morphology to cobblestones. After 4.25% glucose (HG) stimulation, the cells were spindle-shaped, while TAM attenuated high glucose-induced morphological changes (Fig. 2e). The CCK8 results showed that HG reduced mouse peritoneal primary cell viability after 48 h, while TAM significantly restored some of the cell viability (Fig. 2f). Stimulation with 4.25% glucose enhanced the protein expression levels of collagen II (an extracellular matrix protein), α-SMA (a mesenchymal marker), vimentin (a mesenchymal marker), and VEGFA (a proangiogenic cytokine) and decreased the expression of E-cadherin (E-cad) (a mesothelial marker) in mouse primary peritoneal cells (Fig. 2g). Tamoxifen (5 μM) reversed the expression changes of these proteins detected by Western blotting (Fig. 2g, h). The changes in MMT markers were further assessed by IF staining (Additional file 1: Fig. S1a). The amount of the cytokine VEGFA was significantly elevated in the cell supernatant of mouse primary cells, and tamoxifen reduced VEGFA release resulting from high-glucose stimulation (Fig. 2i). EdU and TUNEL results showed that HG reduced proliferation and promoted the apoptosis of mouse peritoneal primary cells, which were moderated by TAM treatment (Additional file 1: Fig. S1b, c).

MeT-5A cells were oval-shaped and differentiated into a shuttle shape upon stimulation with 4.25% glucose for 72 h, while TAM reversed the morphology trend (Fig. 2j). The CCK-8 results indicated that MeT-5A cell viability was reduced under 48 h and 72 h of high-glucose stimulation, effects that were reversed by tamoxifen treatment (Fig. 2k). HG-induced MMT marker protein changes measured by Western Blot (Fig. 2l, m) and immunofluorescence (IF) staining (Additional file 1: Fig. S2a) of MeT-5A cells were palliated by TAM (Fig. 2n). ELISA assay showed that HG promoted VEGFA secretion from MeT-5A cells and TAM treatment reduced VEGFA release. EdU and TUNEL assays showed decreased proliferation and increased apoptosis of peritoneal mesothelial cells in response to high-glucose stimulation, and TAM treatment exerted a protective effect (Additional file 1: Fig. S2b, c).

In conclusion, TAM mitigated high glucose-induced peritoneal fibrosis and protected mouse peritoneal function. TAM preserved cell viability, sustained cell morphology, and suppressed change of MMT marker protein in mouse and human peritoneal cells, respectively, upon high glucose stimulation.

### High glucose promoted H19 expression through ESR1, and TAM repressed this process

**Fig. 3:**
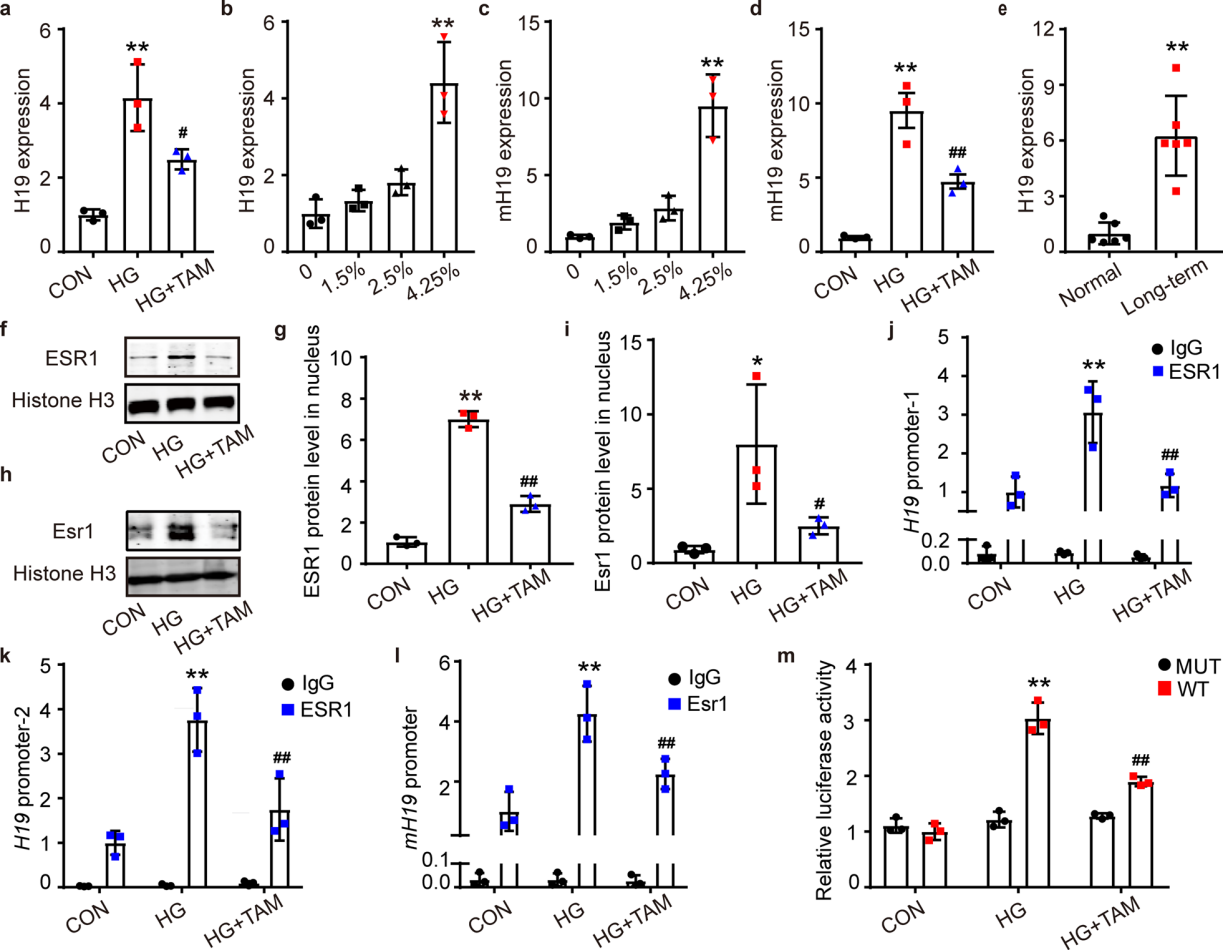
High glucose promoted H19 expression through ESR1, and TAM repressed this process. **a** The expression of H19 in MeT-5A cell lines of the CON, HG, and HG + TAM groups by qRT-PCR. n = 3. **P < 0.01 vs. CON group, ^**#**^*P* < 0.05 vs. HG group. **b** qRT‒PCR to detect the expression of H19 in the MeT-5A cell line under stimulation with different glucose concentrations. n = 3. **P < 0.01 vs. 0% glucose group. **c** mH19 expression in mouse peritoneal primary cells at different glucose concentrations by qRT-PCR. n = 3. **P < 0.01 vs. 0% glucose group. **d** mH19 levels in mouse peritoneal primary cells of the CON, HG, and HG + TAM groups. n = 3. **P < 0.01 vs. CON group, ^**##**^*P* < 0.01 vs. HG group. **e** qRT-PCR to detect H19 expression in the peritoneums of clinical patients. n = 6 samples. **P < 0.01 vs. CON group. **f** Western blot analysis of ESR1 nuclear protein expression in the CON, HG, and HG + TAM groups in the MeT-5A cell line. **g** Relative quantification of ESR1 protein level according to Western Blot images. n = 3. **P < 0.01 vs. CON group, ^**##**^*P* < 0.01 vs. HG group. **h** Western blot analysis of the nuclear expression of Esr1 in mouse peritoneal primary cells in the CON, HG, and HG + TAM groups. **i** Quantification of ESR1 protein level according to Western Blot images in murine peritoneal cells. n = 3. *P < 0.05 vs. CON group, ^**#**^*P* < 0.05 vs. HG group. **j** and** k** ESR1 enrichment levels in the two binding sites of *H19* promoter in MeT-5A cells by ChIP assay. The ChIP assay was used to obtain DNA fragments bound to the ESR1 antibody, followed by the design of primers based on the predicted binding sites for qPCR quantification. Enrichment was normalized to input controls. Primers for ChIP were available in Additional file 1: Table 3. n = 3. **P < 0.01 vs. CON/ESR1 group, ^**##**^*P* < 0.01 vs. HG/ESR1 group. There is no data significance among the IgG group. **l** ESR1 binding sites of the *H19* promoter mice. n = 3. **P < 0.01 vs. CON/Esr1 group, ^**##**^*P* < 0.01 vs. HG/Esr1 group. There is no data significance among the IgG group. **m** Luciferase activity in MeT-5A cells transfected with luciferase reporters containing the wild-type or mutant H19 promoter region. The two binding points were both mutated in the reporter plasmid. The results are shown as the ratio of firefly luciferase activity to Renilla luciferase activity. n = 3. **P < 0.01 vs. CON/WT group, ^**##**^*P* < 0.01 vs. HG/WT group. There is no data significance among the MUT group. Values are the mean ± SD. Two-way ANOVA followed by Tukey’s post-test. *H19* long noncoding RNA H19, *mH19* H19 in mice

We tested several fibrotic disease-related lncRNAs in MeT-5A cells under HG stimulation with or without TAM treatment (Additional file 1: Fig. S3). H19 presented a significant fold change under HG stimulation, and TAM treatment reversed this trend (Fig. 3a). In addition, H19 RNA expression was increased by glucose stimulation in the MeT-5A cell line and murine peritoneal primary cells in a dose-dependent manner (Fig. 3b, c). H19 expression also has a gradual rise in MeT-5A cells and murine peritoneal primary cells with 4.25% glucose stimulation time extending (Additional file 1: Fig. S4). Similar results that TAM reversed the alteration in H19 expression were also found in murine peritoneal primary cells (Fig. 3d). Further, H19 was more highly expressed in the human peritoneum from long-term PDF exposure by qPCR (Fig. 3e).

Western blot analysis showed a marked increase in ESR1 protein in the nucleus, while TAM lessened the nuclear aggregation of ESR1 in the MeT-5A cells (Fig. 3f, g) and murine peritoneal primary cells stimulated with HG (Fig. 3h, i). To investigate how ESR1 regulates H19 RNA expression in the nucleus, we predicted whether the transcription factor ESR1 could bind the promoter region of *H19* in human and mice using the JASPAR and UCSC databases, respectively (Additional file 1: Fig. S5). Then, we verified that ESR1 bound the *H19* promoter region in human and mouse peritoneal cells through ChIP experiments. HG promoted the two binding sites of ESR1 to the *H19* promoter in MeT-5A cells and one site in murine cells, which were reduced by TAM treatment (Fig. 3j-l). The 500 bp upstream of the human H19 transcription start site was cloned into the pSI plasmid with firefly and Renilla luciferases, and two predicted ESR1 binding sites were mutated in the MUT-H19-promoter reporter plasmid. Dual‐luciferase reporter assays showed that HG increased the luciferase activity of the wild-type (WT) H19-promoter reporter plasmid, which was blocked by TAM in MeT-5A cells (Fig. 3m).

### The pro-fibrotic effect of HG was reversed by siH19, while aberrantly elevated H19 exacerbated fibrotic pathological changes in MeT-5A cells

**Fig. 4:**
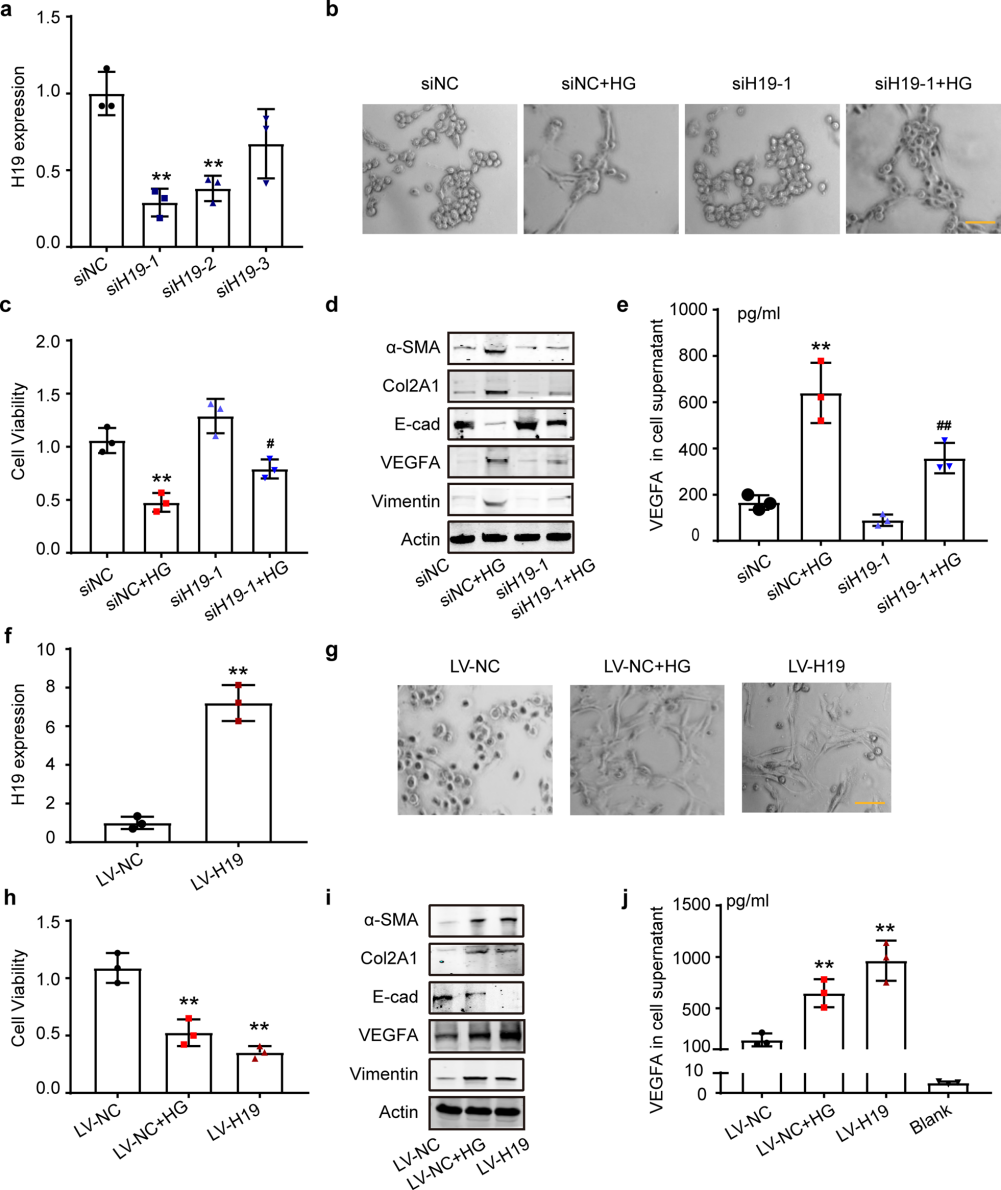
The pro-fibrotic effect of HG was reversed by siH19, while aberrantly elevated H19 exacerbated fibrotic pathological changes in MeT-5A cells. **a** qRT-PCR to validate the efficiency of siH19 in MeT-5A cells. n = 3. **P < 0.01 vs. siNC group. **b** Images of MeT-5A cell morphology after transfection with siNC or siH19. **c** CCK8 assay showed the viability of MeT-5A cells transfected with small interfering RNAs with or without HG stimulation. n = 3. **P < 0.01 vs. siNC group, ^**#**^*P* < 0.05 vs. siNC + HG group. **d** Western blot analysis of EMT and fibrosis markers in MeT-5A cells transfected with siNC or siH19 and stimulated with HG. **e** ELISA to detect the VEGFA protein in the cell supernatant of MeT-5A cells. n = 3. **P < 0.01 vs. siNC group, ^**##**^*P* < 0.01 vs. siNC + HG group. **f** qRT-PCR to validate the efficiency of LV-H19 compared to LV-NC. n = 3. **P < 0.01 vs. LV-NC group. **g** Morphology images of MeT-5A cells infected with LV-H19 or LV-NC. **h** CCK8 assay showing cell viability. n = 3. **P < 0.01 vs. LV-NC group. **i** Western blot analysis of EMT and fibrosis markers. **j** ELISA to detect the VEGFA protein in LV-H19- or LV-NC-infected MeT-5A cell line supernatant. n = 3. **P < 0.01 vs. LV-NC group. Values are the mean ± SD. The pairwise comparison between groups was tested by Dunnett's T3 following One-way ANOVA

To confirm the role of H19 in PD-related fibrosis, we transiently inhibited H19 in MeT-5A cells using small interfering RNAs (siRNAs). First, we verified the interference efficiency and selected siRNA targeting H19-1 (siH19-1) for the following experiments (Fig. 4a). Compared with Met-5A cells transfected with siNC + HG, cells transfected with siH19 alleviated the HG-induced morphological changes, maintained cell viability, relieved the expression of MMT marker molecules, and decreased VEGFA release (Fig. 4b-e). Statistical data analysis of MMT marker protein images acquired by Western Blot showed no significance of E-cad protein level between the siH19-1+ HG group and siNC + HG (Additional file 1: Fig. S6a). Further, compared with the siNC group, the siH19-1 group protein changes were also not statistically significant (Additional file 1: Fig. S6a). SiH19 reversed HG-induced MMT marker protein expression detected by IF staining (Additional file 1: Fig. S6b) and restored cell proliferation ability, and reduced apoptosis (Additional file 1: Fig. S6c, d).

MeT-5A cells infected with LV-H19 overexpressed H19 sevenfold and presented severe morphological alterations compared to LV-NC (Fig. 4f, g). Similar to the LV-NC+ HG group, MeT-5A cells infected with LV-H19 were hyperextended, and fusiform with long tentacles. H19 overexpression resulted in a significant decrease in cell viability, a marked induction of MMT marker protein change, and an increase in VEGFA release (Fig. 4h-j). Analysis of Western Blot images and IF merge images of MMT marker proteins also supported the above conclusions (Additional file 1: Fig. S7a, b). In addition, H19 overexpression led to a pronounced loss of proliferative capacity and elevated apoptosis (Additional file 1: Fig. S7c, d).

### Interference with H19 inhibited PD-related peritoneal fibrosis in the mouse

**Fig. 5:**
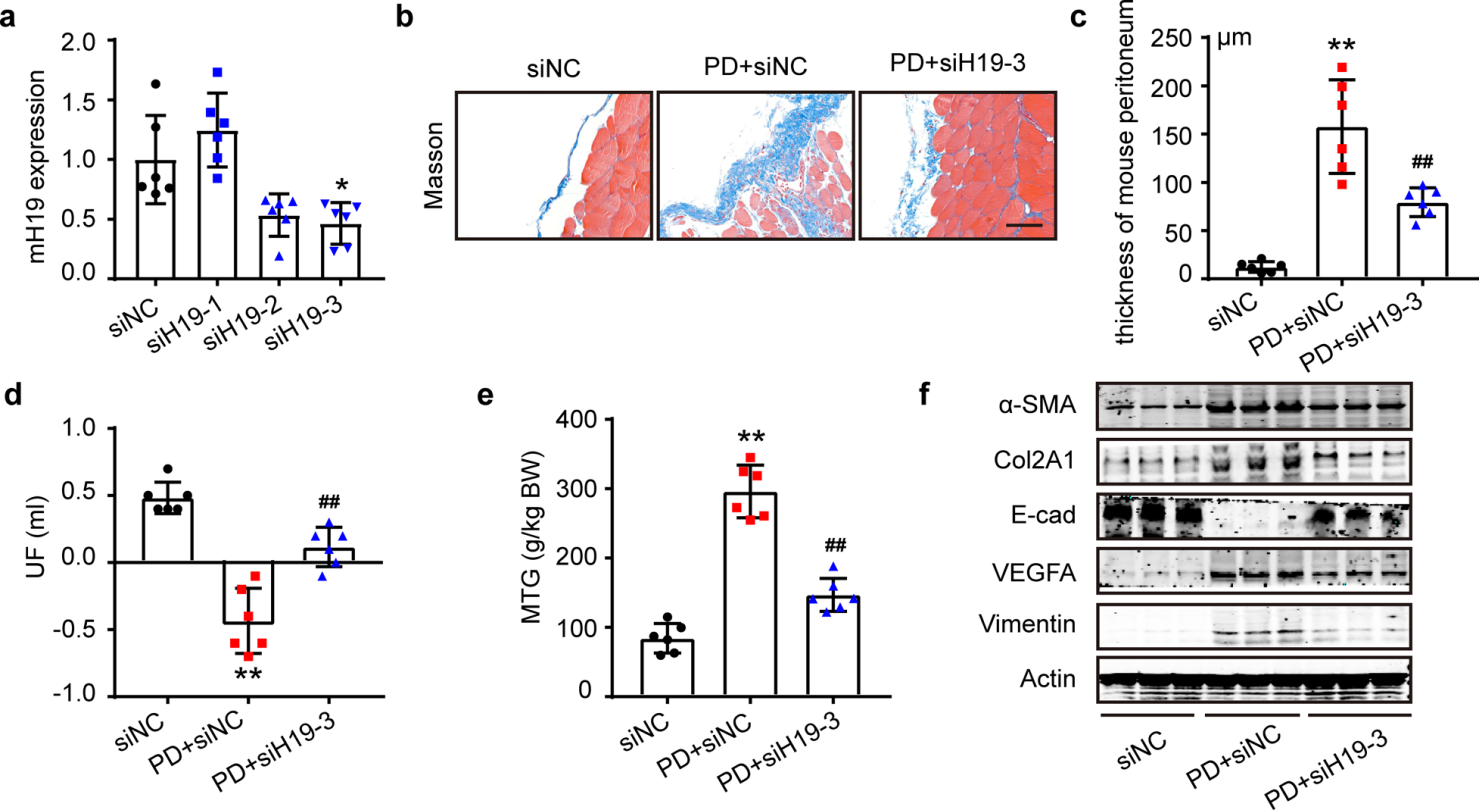
Interference with H19 inhibited PD-related peritoneal fibrosis in the mouse. **a** qRT-PCR to validate the efficiency of siRNAs targeting mouse H19. The nanomaterial-encapsulated modified siRNAs were injected into the peritoneal cavity of mice for 24 h. Then, the peritoneal cells were extracted by EDTA-trypsin digestion. Total RNA was extracted by the phenol/chloroform method, followed by qRT-PCR. We chose siH19-3 for the following experiment. n = 6 mice. Values were performed normality test followed by Dunn’s comparisons test. **b** Masson-stained images showed peritoneal collagen deposition in mice. Scale bars = 100 μm. **c** Relative quantification of mouse peritoneal thickness by ImageJ. n = 6. **P < 0.01 vs. siNC group. ^**##**^*P* < 0.01 vs. PD + siNC group. **d** and **e** Peritoneal function test of ultrafiltration failure (UF) measurements and glucose transfer (MTG) in the mouse. n = 6. **P < 0.01 vs. siNC group. ^**##**^*P* < 0.01 vs. PD + siNC group. **f** Western blot analysis of EMT and fibrosis markers in mouse peritoneal tissues. Values are the mean ± SD. The pairwise comparison between groups was tested by Dunnett's T3 following One-way ANOVA (c-e)

To examine whether interfering with H19 could restrain high glucose-induced peritoneal fibrosis in mice, we injected PDF mixed with small interfering RNA targeting mouse H19 for treatment or siNC as a negative control. 2′-O-Me-modified small interfering RNAs (0.5 mg/kg) were wrapped with nanoliposomes in vitro and incubated at room temperature for 15 min. Then, the mixture was diluted with PDF (0.1 ml/g) or saline and injected intraperitoneally daily. We chose siH19-3 for the following experiments according to the in vivo efficiency (Fig. 5a). Histopathological measurements revealed less collagen deposition in the PD + siH19-3 group than in the PD + siNC group (Fig. 5b, c). Additionally, siH19 treatment preserved peritoneal function compared to the PD + siNC group by increasing the UF rate and reducing peritoneal solute transport (Fig. 5d, e). Western blot assays of murine peritoneal tissues showed that siH19 restored the PDF-induced MMT process by reducing mesenchymal protein collagen II, α-SMA, vimentin, and VEGFA and increasing mesothelial marker E-cad (Fig. 5f).

### H19 facilitated peritoneal fibrosis by binding p300 and acetylating H3K27 in the VEGFA promoter region

**Fig. 6:**
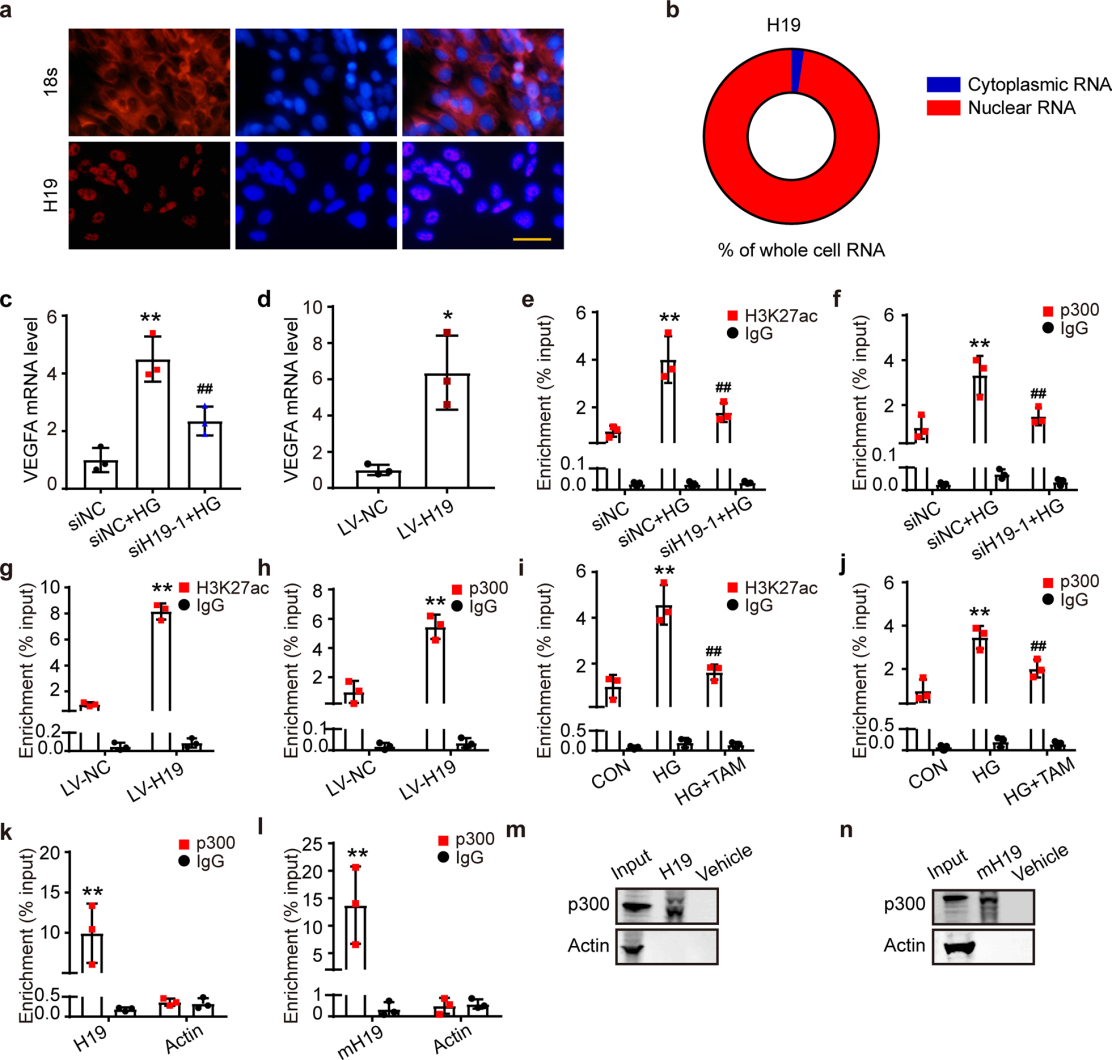
H19 facilitated peritoneal fibrosis by binding p300 and acetylating H3K27 in the VEGFA promoter region. **a** RNA FISH showed the localization of H19 in MeT-5A cells. An 18 s probe was used in the positive control. Scale bars = 100 μm. **b** Quantification of H19 in the cytoplasm and nucleus by cytoplasmic and nuclear RNA isolation experiments. **c** qRT-PCR measured the VEGFA mRNA levels in MeT-5A cells transfected with siH19 or siNC and stimulated with HG. n = 3. **P < 0.01 vs. siNC group, ^**##**^*P* < 0.01 vs. siNC + HG group. **d** qRT-PCR to test LV-NC- or LV-H19-infected MeT-5A cells. n = 3. **P < 0.01 vs. LV-NC group. **e**–**f** ChIP assay to detect H3K27ac and p300 binding to the *VEGFA* promoter region in H19 knockdown MeT-5A cells. n = 3. **P < 0.01 vs. siNC group, ^**##**^*P* < 0.01 vs. siNC + HG group. **g** and **h** ChIP assay to detect H3K27ac and p300 binding to the *VEGFA* promoter region in LV-H19 overexpression MeT-5A cells. n = 3. **P < 0.01 vs. LV-NC group. **i** and **j** ChIP assays were performed to detect H3K27ac and p300 binding to the *VEGFA* promoter region in MeT-5A cells under HG stimulation and TAM treatment. n = 3. **P < 0.01 vs. CON group, ^**##**^*P* < 0.01 vs. HG group. **k** and **l** RIP assay showing p300-bound H19 in MeT-5A cells and mouse peritoneal primary cells. RNA enrichment was determined by qRT-PCR and normalized to the input control. IgG and Actin RNA were used as negative controls. n = 3. **P < 0.01 vs. H19/p300 group. There is no data significance between Actin/p300 and Actin/IgG group. Two-way ANOVA was performed followed by Dunnett's T3. **m** and **n** RNA pull-down was used to analyze protein binding to H19. RNA-binding proteins were enriched and analyzed by Western blotting. All experiments were performed three times. Values are the mean ± SD. Two-way ANOVA was performed followed by Tukey's test (e-i)

The mechanism by which H19 promotes PD-associated fibrosis has not been investigated and aroused our curiosity. First, we determined the nuclear localization of H19 in MeT-5A cells by RNA FISH (Fig. 6a). Moreover, we quantified the distribution of H19 by cytoplasmic and nuclear RNA isolation, and the results also showed that 97% of H19 was located in the nucleus (Fig. 6b). Thus, H19 exerted its function mostly in the nucleus of MeT-5A cells. VEGFA is an important cytokine contributing to ultrafiltration failure in peritoneal dialysis fibrosis, which leads to pathological proliferation of peritoneal vessels as well as peritoneal fibrosis with reduced osmotic pressure in the clinic. qPCR results revealed that interference with H19 suppressed HG-induced elevated levels of VEGFA mRNA, and H19 overexpression increased the level of VEGFA mRNA in MeT-5A cells (Fig. 6c, d). This finding implied that H19 might affect *VEGFA* transcription.

H3K27ac in the *VEGFA* promoter region is considered an essential factor in regulating *VEGFA* transcription, where p300 acts as a transcriptional coactivator with histone acetyltransferase activity. We knocked down p300 expression in MeT-5A cells with siRNAs and fewer VEGFA mRNA levels were detected by qPCR (Additional file 1: Fig. S8a, b).

We detected enrichment changes in H3K27ac and p300 in the *VEGFA* promoter through ChIP experiments on MeT-5A cells. HG increased the H3K27ac and p300 enrichment in the *VEGFA* promoter, which was reversed by siH19-1 (Fig. 6e, f). Overexpression of H19 in MeT-5A cells advanced H3K27ac and p300 enrichment in the *VEGFA* promoter (Fig. 6g, h). Subsequently, we demonstrated that HG promoted p300 and H3K27ac enrichment in the *VEGFA* promoter in MeT-5A cells, while TAM treatment suppressed this enrichment (Fig. 6i, j).

In the nucleus, LncRNAs can guide chromatin modifiers and various transcriptional regulators to DNA to repress or activate gene expression. The *CatRAID* omics v2.1 database predicted H19 and p300 binding loci based on hydrogen bonding, van der Waals forces, and the secondary structure of RNA in humans and mice (Additional file 1: Fig. S9a, b). To validate the direct binding between H19 and p300, we performed RNA immunoprecipitation (RIP) to pull down endogenous RNAs associated with p300 and assessed them via qPCR analysis in the human peritoneal mesothelial cell line, MeT-5A and mouse peritoneal primary cells (Fig. 6k, l). The specific association between H19 and p300 was further validated by affinity pull-down using in vitro transcribed biotin-labelled H19. Western blot analysis showed binding between p300 and H19 (Fig. 6m, n).

### The pro-fibrotic effect of H19 could be partially blocked by interfering with VEGFA

**Fig. 7:**
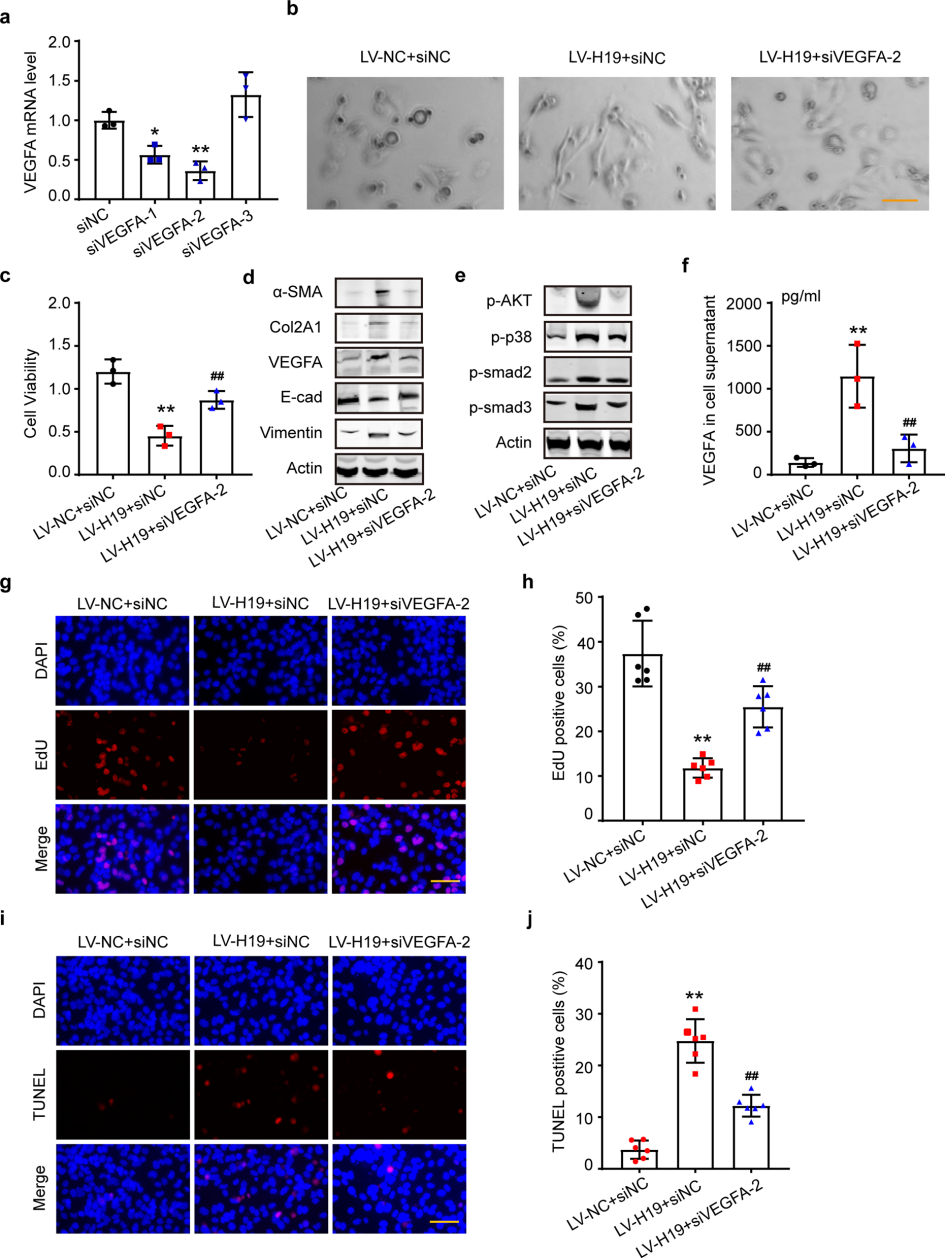
The pro-fibrotic effect of H19 can be partially blocked by interfering with VEGFA **a** Validation of the siVEGFA efficiency in MeT-5A cells by qRT-PCR. n = 3. *P < 0.05, **P < 0.01 vs. siNC group. **b** Images showing the morphology of LV-H19- or LV-NC-infected MeT-5A cells transfected with siVEGFA-2 or siNC. **c** CCK8 showing cell viability. n = 3. **P < 0.01 vs. LV-NC + siNC group, ^**##**^*P* < 0.01 vs. LV-H19 + siNC group. **d** and **e** Western blot analysis of EMT markers and pathways activated in the EMT process and fibrosis. **f** ELISA showing VEGFA in the cell supernatant. n = 3. **P < 0.01 vs. LV-NC + siNC group, ^**##**^*P* < 0.01 vs. LV-H19 + siNC group. **g** and **h** EdU assay for the proliferation of LV-H19- or LV-NC-infected MeT-5A cells and quantification of EdU-positive cells. Scale bars = 100 μm. n = 3. **P < 0.01 vs. LV-NC + siNC group, ^**##**^*P* < 0.01 vs. LV-H19 + siNC group. **i** and **j** TUNEL Bright Red test showing apoptosis and quantification of TUNEL-positive cells of LV-H19- or LV-NC-infected MeT-5A cells. n = 3. **P < 0.01 vs. LV-NC + siNC group, ^**##**^*P* < 0.01 vs. LV-H19 + siNC group. Scale bars = 100 μm. n = 3. One-way ANOVA was performed followed by Dunnett's T3

To investigate whether H19 exerted its pro-fibrotic functions through VEGFA, we transfected siVEGFA into LV-H19-infected Met-5A cells. siVEGFA-2 had a significant interference efficiency against VEGFA (Fig. 7a). We chose siVEGFA-2 for the following functional rescue experiments. LV-H19+ siVEGFA-2 relieved cellular morphological changes and increased cell viability compared to LV-H19+ siNC (Fig. 7 b, c). Western blot analysis showed that fibrosis-related protein changes and H19-induced activation pathways were disrupted by siVEGFA-2 (Fig. 7d, e), but Western blot statistical analysis showed E-cad and p-smad3 protein changes had no statistical significance (Additional file 1: Fig. S10a, b). There was less VEGFA in the cell supernatant upon comparing LV-H19 + siVEGFA-2 with LV-H19 + siNC (Fig. 7f). EdU and TUNEL assays showed that siVEGFA relieved H19-induced cell apoptosis and restored proliferation ability (Fig. 7g-j).

### Roles of ESR1 and H19 RNA in predicting peritoneal function

**Fig. 8:**
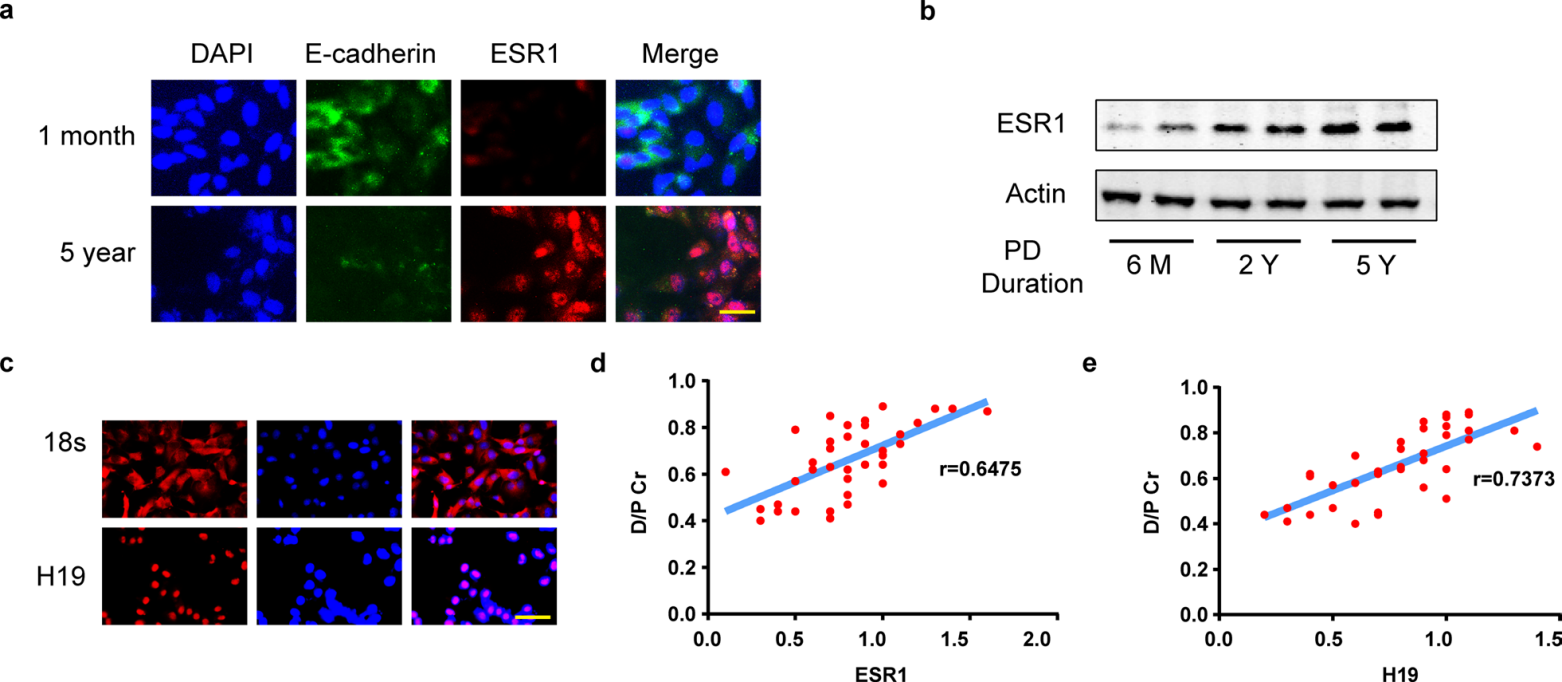
Roles of ESR1 and H19 RNA in predicting peritoneal function. **a** HPMCs were cultured from PD effluent of two patients (1-month duration and 5-year duration). E-cadherin and ESR1 expressions were detected by immunofluorescence. Scale Bar = 20 μm. **b** Images of Western Blot analysis showing ESR1 protein levels of PD effluent cell lysates from patients (PD duration: 6 months, 2 years, and 5 years). **c** Localization of H19 in HPMCs was presented by RNA FISH. Scale Bar = 50 μm. **d** and **e** A positive linear correlation between ESR1 (or H19) RNA levels in human exfoliated mesothelial cells and D/P Cr. Pearson correlation was conducted. *HPMCs* human peritoneal mesothelial cells, *D/P* Cr dialysate/plasma ratio of creatinine

In order to investigate expressions of ESR1 and long non-coding RNA H19 in patients undergoing regular peritoneal dialysis, we extracted human peritoneal mesothelial cells 37 patients’ overnight PD effluent of 37 patients. Exfoliated human mesothelial cells in drained PDF from every patient were collected. Compared to short-term PD, long-term exposure to glucose led to the loss of mesothelial marker E-cadherin and the gain of high expression of ESR1 in the nucleus as immunofluorescence (Fig. 8a). ESR1 protein extracted from exfoliated human mesothelial cells in drained PDF was increased in a PD course time-dependent way (Fig. 8b). In addition, consistent with the previous results of MeT-5A, H19 was clearly labeled in the nucleus by the FISH method, indicating its distribution in the nuclei of human peritoneal mesothelial cells (Fig. 8c).

Total RNA was extracted to analyze the correlation between ESR1 (or H19) RNA levels and sex, age, time of PD duration, and peritoneal transport function (Additional file 1: Table S1b). ESR1 and H19 RNA levels were calculated by CT values relative to Actin level (2^^-ΔCt^ method) respectively. There was no statistical difference in ESR1 (or H19) RNA levels between the female and male groups. Statistical results also suggested that ESR1 (or H19) RNA levels had nothing to do with age. ESR1 (or H19) RNA levels were higher in patients with PD duration over 60 months than in those durations less than 60 months. Further, patients with high transport (H) and high average transport (HA) expressed up-regulated levels than those with low transport (L) and low average transport (LA) (Additional file 1: Table S1b). Therefore, we further analyzed the correlation between ESR1 (or H19) RNA levels and the D/P Cr (dialysate/plasma ratio of creatinine). It was suggested that ESR1 (or H19) RNA levels in cast-off human mesothelial cells from drained peritoneal dialysis effluent had a positive linear correlation with high peritoneal solute transfer rate (Fig. 8d, e).

However, the clinical sample size was small, so it was necessary to expand the sample size for in-depth analysis in different PD centers.

## Discussion

The nonphysiological composition of the peritoneal dialysis fluid (PDF) induces mesothelial cells to undergo a mesothelial-to-mesenchymal transition and release extracellular matrix and pro-fibrotic cytokines [27]. Signs of peritoneal fibrosis, causing peritoneal membrane dysfunction, are detected in 50 to 80% of patients within one to 2 years and hinder its utilization in clinical treatment [10]. Tamoxifen is a selective oestrogen receptor modulator that is able to affect multiple tissues in the body. Of particular interest are several reports describing tamoxifen’s efficacy in promoting the regression of different fibrotic diseases [28]. Previous studies proved that tamoxifen potently inhibited mesothelial-mesenchymal transition, stromal fibrosis, and neoangiogenic processes [29, 30]. Our study demonstrated that TAM played a protective role in PDF-induced fibrosis *in viv*o and in vitro.

Reducing the ESR1 level in the MeT-5A cell line led to anti-fibrotic effects against HG stimulation (Additional file 1: Fig. S11). Furthermore, our study showed a significant increase in ESR1 in the nucleus by HG stimulation, which was reversed by tamoxifen, implying that tamoxifen exerted its protective effect against fibrosis by blocking ESR1 translocation into the nucleus. At the same time, we tested ESR2 in Met-5A cells and found that ESR2 total protein and mRNA levels were decreased in a glucose-dependent manner(0, 1.5%, 2.5%, 4.25%) (Additional file 1: Fig. S12a, b), while tamoxifen did not affect its nuclear expression (Additional file 1: Fig. S12c). This result is consistent with the fact that ESR1 and ESR2 are antagonistic in physiological functions, ESR1 is generally known as a fibrogenic factor, and ESR2 is an anti-fibrotic factor.

Although LncRNAs do not possess the ability to encode proteins, they are essential regulators of cellular signalling and relevant diseases [15]. We screened several common fibrosis-associated LncRNAs [15, 31] and confirmed that H19 was consistent with this prediction. H19 RNA level was reduced by siRNA targeting ESR1 in MeT-5A cells (Additional file 1: Fig. S13). In previous studies, Klein and Yasmin reported that H19 was an oestrogen receptor-targeted lncRNA [16, 32], and ESR1 bound the H19 locus by in vivo ChIP-Seq experiments [33–35]. Our study confirmed this conclusion by ChIP and dual-luciferase reporter assays.

In previous studies, H19 was found to acts as a miRNA sponge to modulate fibrosis [36, 37]. However, H19 is predominantly localized in the nucleus of peritoneal cells, which is obviously inconsistent with this mechanism. Because LncRNAs can function as chromatin modifiers, regulators, or enhancers in the nucleus to repress and/or activate gene expression and H19 regulated the level of VEGFA mRNA, we hypothesized that H19 affected *VEGFA* transcription in the nucleus. The pivotal factor affecting *VEGFA* transcription is the essential transcriptional coactivator p300, which forms the hypoxia-inducible factor 1 (HIF-1α)/p300 complex [38], modulates H3K27ac, and is a marker of active transcription [39, 40]. ChIP assays revealed that H19 promoted p300 and H3K27ac enrichment in the *VEGFA* promoter region in our study. It was reported that LncRNA interacts with p300 and regulates HAT activity [21, 23]. We confirmed the binding of H19 and p300 in the human peritoneal cell line, MeT-5A and mouse primary peritoneal cells by RIP and RNA pull-down assays.

Finally, we confirmed the profibrotic effect of H19 was partially blocked by siVEGFA. siVEGFA relieved the alteration of the MMT process marker molecules and reduced the activation of fibrotic pathways induced by H19. The PI3K/Akt, MAPK/p38, and TGF-β/smad pathways are highly associated with peritoneal fibrosis as reported in previous studies. We presumed that H19 promoted VEGFA release and led to the activation of these pathways by autocrine secretion.. We treated MeT-5A cells with Axitinib (2 nM) (HY-10065, MedChemExpress, USA) to inhibit VEGFA receptors. Western Blot analysis results indicated that H19 overexpression promoted the MMT process while inhibiting VEGFA/VEGFR axis inhibiting the MMT process of human peritoneal mesothelial cells (Additional file 1: Fig. S15). The results illustrated that H19 exerted pro-fibrotic effects through VEGFA and neutralizing VEGFA could suppress the effects.

### Supplementary Information

**Additional file 1: **Complete materials and methods. 1. Human peritoneum specimens. 2. PDF-induced peritoneal fibrosis in mice and tamoxifen administration. 3. Peritoneal equilibrium test. 4. Intraperitoneal delivery of liposomal siRNAs. 5. Cell culture. 6. Peritoneal histology and immunofluorescence. 7. Cell viability. 8. Transfection of siRNAs in MeT-5A cells. 9. Construction of lentivirus-infected stable cell lines. 10. RNA extraction and quantitative real-time PCR (qRT‒PCR). 11. Western blot analysis. 12. ELISA. 13. Detection of cell proliferation by EdU. 14. Detection of cell apoptosis by TUNEL. 15. Chromatin immunoprecipitation (ChIP). 16. Dual‐luciferase reporter assay. 17. Cytoplasmic and nuclear RNA extraction. 18. RNA immunoprecipitation (RIP). 19. RNA FISH. 20. RNA pull-down assay. 21. Statistical analysis. **Table S1.** Patients’ information. **Table S2.** Primers for qRT-PCR. **Table S3.** Primers used in chromatin immunoprecipitation assay. **Figure S1.** Immunofluorescence merge images, EdU, and TUNEL staining of murine primary cells. **Figure S2.** Immunofluorescence merge images, EdU, and TUNEL staining of MeT-5A cells. **Figure S3. **Fibrotic disease-related LncRNA expression changes in MeT-5A cells under HG stimulation and TAM treatment. **Figure S4.** H19 expression changes. **Figure S5.** ESR1 binding site of *H19* promoter region by JASPAR and UCSC databases. **Figure S6.** Effects of siH19 against HG stimulated-MMT of MeT-5A cells. **Figure S7. **Overexpression H19 Promoted MMT of MeT-5A cells. **Figure S8.** Reducing p300 in MeT-5A cells suppressed VEGFA mRNA level. **Figure S9.** Predictions of H19 and p300 binding in human and mice. **Figure S10.** Statistical analysis of MMT protein changes in LV-H19 infected MeT-5A cells. **Figure S11.** HG-induced pro-fibrotic effect was reversed by siESR1 in MeT-5A. **Figure S12.** ESR2 total protein and mRNA levels were decreased in a glucose-dependent manner, while tamoxifen did not affect its nuclear expression in MeT-5A cells. **Figure S13.** H19 RNA level was decreased by siRNA targeting ESR1 in MeT-5A cells. **Figure S14.** Angiogenesis markers in mouse models. **Figure S15.** Western Blot analysis of MeT-5A cells treated with Axitinib.

## Data Availability

All the data used for this study are presented in the paper or the Additional file 1: Materials. The datasets used and/or analyzed during the current study are available from the corresponding author on reasonable request.

## Abbreviations

PD: Peritoneal dialysis
TAM: Tamoxifen
ESR1: Estrogen receptor 1
ChIP: Chromatin immunoprecipitation
MMT: Mesothelial-mesenchymal transition
HPMCs: Human peritoneal mesothelial cells
PSTR: Peritoneal solute transfer rate
RIP: RNA immunoprecipitation
VEGFA: Vascular endothelial growth factor A
PDF: Peritoneal dialysis fluid
SERM: Selective estrogen receptor modulator
LncRNA: Long noncoding RNA
HAT: Histone acetyltransferase
CAPD: Continuous ambulatory peritoneal dialysis
IF: Immunofluorescence
UF: Ultrafiltration rate
MTG: Mass transfer of glucose
WT: Wild-type
siRNAs: Small interfering RNAs
D/P Cr: Dialysate/plasma ratio of creatinine

## References

[1] Teitelbaum I (2021). Peritoneal dialysis. N Engl J Med.

[2] Trionfetti F, Marchant V, González-Mateo GT (2023). Novel aspects of the immune response involved in the peritoneal damage in chronic kidney disease patients under dialysis. Int J Mol Sci.

[3] Krediet RT (2021). Acquired decline in ultrafiltration in peritoneal dialysis: the role of glucose. J Am Soc Nephrol.

[4] Leung JC, Chan LY, Li FF (2005). Glucose degradation products downregulate ZO-1 expression in human peritoneal mesothelial cells: the role of VEGF. Nephrol Dial Transpl.

[5] Selgas R, Bajo A, Jiménez-Heffernan JA (2006). Epithelial-to-mesenchymal transition of the mesothelial cell–its role in the response of the peritoneum to dialysis. Nephrol Dial Transpl.

[6] Bartosova M, Schaefer B, Bermejo JL (2018). Complement activation in peritoneal dialysis-induced arteriolopathy. J Am Soc Nephrol.

[7] Pérez-Lozano ML, Sandoval P, Rynne-Vidal A, Aguilera A, Jiménez-Heffernan JA, Albar-Vizcaíno P (2013). Functional relevance of the switch of VEGF receptors/co-receptors during peritoneal dialysis-induced mesothelial to mesenchymal transition. PLoS ONE.

[8] He Q, Wen L, Wang L, Zhang Y, Yu W, Zhang F (2020). miR-15a-5p suppresses peritoneal fibrosis induced by peritoneal dialysis via targeting VEGF in rats. Ren Fail.

[9] Krediet RT, Struijk DG (2013). Peritoneal changes in patients on long-term peritoneal dialysis. Nat Rev Nephrol.

[10] Jagirdar RM, Bozikas A, Zarogiannis SG, Bartosova M, Schmitt CP, Liakopoulos V (2019). Encapsulating peritoneal sclerosis: pathophysiology and current treatment options. Int J Mol Sci.

[11] de Sousa E, del Peso-Gilsanz G, Bajo-Rubio MA, Ossorio-González M, Selgas-Gutiérrez R (2012). Encapsulating peritoneal sclerosis in peritoneal dialysis a review and European initiative for approaching a serious and rare disease. Nefrologia.

[12] Loureiro J, Sandoval P, del Peso G (2013). Tamoxifen ameliorates peritoneal membrane damage by blocking mesothelial to mesenchymal transition in peritoneal dialysis. PLoS ONE.

[13] Fontaine C, Morfoisse F, Tatin F (2020). The impact of estrogen receptor in arterial and lymphatic vascular diseases. Int J Mol Sci.

[14] Cech TR, Steitz JA (2014). The noncoding RNA revolution-trashing old rules to forge new ones. Cell.

[15] Yang Z, Jiang S, Shang J (2019). LncRNA: Shedding light on mechanisms and opportunities in fibrosis and aging. Ageing Res Rev.

[16] Li X, Liu R, Yang J (2017). The role of long noncoding RNA H19 in gender disparity of cholestatic liver injury in multidrug resistance 2 gene knockout mice. Hepatology.

[17] Omura J, Habbout K, Shimauchi T (2020). Identification of long noncoding RNA H19 as a new biomarker and therapeutic target in right ventricular failure in pulmonary arterial hypertension. Circulation.

[18] Liu R, Li X, Zhu W (2019). Cholangiocyte-derived exosomal long noncoding RNA H19 promotes hepatic stellate cell activation and cholestatic liver fibrosis. Hepatology.

[19] Xiao Y, Liu R, Li X (2019). Long Noncoding RNA H19 contributes to cholangiocyte proliferation and cholestatic liver fibrosis in biliary atresia. Hepatology.

[20] Ogryzko VV, Schiltz RL, Russanova V, Howard BH, Nakatani Y (1996). The transcriptional coactivators p300 and CBP are histone acetyltransferases. Cell.

[21] Ortega E, Rengachari S, Ibrahim Z (2018). Transcription factor dimerization activates the p300 acetyltransferase. Nature.

[22] Raisner R, Kharbanda S, Jin L (2018). Enhancer activity requires CBP/P300 bromodomain-dependent histone H3K27 acetylation. Cell Rep.

[23] Bose DA, Donahue G, Reinberg D, Shiekhattar R, Bonasio R, Berger SL (2017). RNA binding to CBP stimulates histone acetylation and transcription. Cell.

[24] Yang X, Bao M, Fang Y, Yu X, Ji J, Ding X (2021). STAT3/HIF-1α signaling activation mediates peritoneal fibrosis induced by high glucose. J Transl Med.

[25] Shi Y, Li J, Chen H (2022). Inhibition of EZH2 suppresses peritoneal angiogenesis by targeting a VEGFR2/ERK1/2/HIF-1α-dependent signaling pathway. J Pathol.

[26] Yang X, Yan H, Jiang N (2020). IL-6 trans-signaling drives a STAT3-dependent pathway that leads to structural alterations of the peritoneal membrane. Am J Physiol Renal Physiol.

[27] Loureiro J, Aguilera A, Selgas R (2011). Blocking TGF-β1 protects the peritoneal membrane from dialysate-induced damage. J Am Soc Nephrol.

[28] Dellê H, Rocha JR, Cavaglieri RC, Vieira JM, Malheiros DM, Noronha IL (2012). Antifibrotic effect of tamoxifen in a model of progressive renal disease. J Am Soc Nephrol.

[29] Elliot S, Periera-Simon S, Xia X (2019). MicroRNA let-7 downregulates ligand-independent estrogen receptor-mediated male-predominant pulmonary fibrosis. Am J Respir Crit Care Med.

[30] Wilson RB, Archid R, Reymond MA (2020). Reprogramming of mesothelial-mesenchymal transition in chronic peritoneal diseases by estrogen receptor modulation and TGF-β1 inhibition. Int J Mol Sci.

[31] Li J, Cao LT, Liu HH, Yin XD, Wang J (2020). Long non coding RNA H19: an emerging therapeutic target in fibrosing diseases. Autoimmunity.

[32] Keniry A, Oxley D, Monnier P (2012). The H19 lincRNA is a developmental reservoir of miR-675 that suppresses growth and Igf1r. Nat Cell Biol.

[33] Adriaenssens E, Lottin S, Dugimont T (1999). Steroid hormones modulate H19 gene expression in both mammary gland and uterus. Oncogene.

[34] Klein RH, Stephens DN, Ho H (2016). Cofactors of LIM domains associate with estrogen receptor α to regulate the expression of noncoding RNA H19 and corneal epithelial progenitor cell function. J Biol Chem.

[35] Vasquez YM, Nandu TS, Kelleher AM, Ramos EI, Gadad SS, Kraus WL (2020). Genome-wide analysis and functional prediction of the estrogen-regulated transcriptional response in the mouse uterus†. Biol Reprod.

[36] Hou J, Wang L, Wu Q (2018). Long noncoding RNA H19 upregulates vascular endothelial growth factor A to enhance mesenchymal stem cells survival and angiogenic capacity by inhibiting miR-199a-5p. Stem Cell Res Ther.

[37] Sun B, Ding Y, Jin X, Xu S, Zhang H (2019). Biosci Rep.

[38] Ma L, Gao Z, Wu J (2021). Co-condensation between transcription factor and coactivator p300 modulates transcriptional bursting kinetics. Mol Cell.

[39] Ghosh AK, Shanafelt TD, Cimmino A (2009). Aberrant regulation of pVHL levels by microRNA promotes the HIF/VEGF axis in CLL B cells. Blood.

[40] Reece KM, Richardson ED, Cook KM (2014). Epidithiodiketopiperazines (ETPs) exhibit in vitro antiangiogenic and in vivo antitumor activity by disrupting the HIF-1α/p300 complex in a preclinical model of prostate cancer. Mol Cancer.

