# Supplementary material for: Tamoxifen exerts anti-peritoneal fibrosis effects by inhibiting H19-activated VEGFA transcription
Source: J Transl Med. 2023 Sep 11;21:614. doi: 10.1186/s12967-023-04470-3 (PMC10494369; doi:10.1186/s12967-023-04470-3)
Supplement: Supplementary file 1 — Additional file 1: Complete materials and methods. 1. Human peritoneum specimens. 2. PDF-induced peritoneal fibrosis in mice and tamoxifen administration. 3. Peritoneal equilibrium test. 4. Intraperitoneal delivery of liposomal siRNAs. 5. Cell culture. 6. Peritoneal histology and immunofluorescence. 7. Cell viability. 8. Transfection of siRNAs in MeT-5A cells. 9. Construction of lentivirus-infected stable cell lines. 10. RNA extraction and quantitative real-time PCR (qRT‒PCR). 11. Western blot analysis. 12. ELISA. 13. Detection of cell proliferation by EdU. 14. Detection of cell apoptosis by TUNEL. 15. Chromatin immunoprecipitation (ChIP). 16. Dual‐luciferase reporter assay. 17. Cytoplasmic and nuclear RNA extraction. 18. RNA immunoprecipitation (RIP). 19. RNA FISH. 20. RNA pull-down assay. 21. Statistical analysis. Table S1. Patients’ information. Table S2. Primers for qRT-PCR. Table S3. Primers used in chromatin immunoprecipitation assay. Figure S1. Immunofluorescence merge images, EdU, and TUNEL staining of murine primary cells. Figure S2. Immunofluorescence merge images, EdU, and TUNEL staining of MeT-5A cells. Figure S3. Fibrotic disease-related LncRNA expression changes in MeT-5A cells under HG stimulation and TAM treatment. Figure S4. H19 expression changes. Figure S5. ESR1 binding site of H19 promoter region by JASPAR and UCSC databases. Figure S6. Effects of siH19 against HG stimulated-MMT of MeT-5A cells. Figure S7. Overexpression H19 Promoted MMT of MeT-5A cells. Figure S8. Reducing p300 in MeT-5A cells suppressed VEGFA mRNA level. Figure S9. Predictions of H19 and p300 binding in human and mice. Figure S10. Statistical analysis of MMT protein changes in LV-H19 infected MeT-5A cells. Figure S11. HG-induced pro-fibrotic effect was reversed by siESR1 in MeT-5A. Figure S12. ESR2 total protein and mRNA levels were decreased in a glucose-dependent manner, while tamoxifen did not affect its nuclear expression in MeT-5A cells. Figure S13. H19 RNA level was decre [file 12967_2023_4470_MOESM1_ESM.docx]

**Supplementary materials and methods.**

**Human peritoneum specimens** Human peritoneum specimens were collected from Shanghai Changhai Hospital. Six normal peritoneal tissues were obtained from patients receiving catheterization for PD. Six long-term PD samples were obtained from patients receiving extubating procedures who suffered peritoneal ultrafiltration failure caused by peritoneal fibrosis and whose PD durations were all over five years. Patients’ information was supplied in Supplementary Table S1A. All patients were informed and consented to the procedure. The Shanghai Changhai Hospital Ethics Committee approved our study (CHBC2020-096)**.**

**PDF-induced peritoneal fibrosis in mice and** **tamoxifen administration** All animal experiments followed the guidelines and protocols of the Animal Ethics Committee of the Naval Medical University. Male C57B/L6 mice (25-30 g) aged eight weeks were purchased from Cavens Lab Animal Co. (Changzhou, China). All animals were housed in specific-pathogen-free (SPF) animal rooms at 25 °C with 12 h light/day and free access to water and food. A mouse model of PD-induced peritoneal fibrosis was established by a daily intraperitoneal injection of 4.25% high-glucose PDF (Baxter, Shanghai, China). We mixed the peritoneal dialysis fluid with 40 mM methylglyoxal (Sigma‒Aldrich, St. Louis, MO) to accelerate fibrosis for 2 weeks [1, 2]. We randomly divided 18 mice into three groups (n = 6 mice): the CON group, the PD group, and the PD+TAM group. The control group received a daily intraperitoneal injection of saline (0.1 ml/g, administered in the lower right quadrant). The PD group received an equal volume of 4.25% PDF containing methylglyoxal daily. Mice in the PDF+TAM group received a daily intraperitoneal injection of PDF containing methylglyoxal and tamoxifen citrate (MCE, HY-13757) treatment, 10 mg/kg, daily, by gavage [3]. On Day 15, peritoneal function tests were performed. Then, the mice were anaesthetized with pentobarbital sodium, and peritoneal samples were harvested from the left half of the abdomen after being sacrificed by cervical dislocation.

**Peritoneal equilibrium test** We performed a peritoneal function test by injecting 0.1 ml/g body weight of 4.25% PDF intraperitoneally [3-5]. After 2 hours, the remaining peritoneal dialysis fluid in the abdominal cavity was aspirated. The UF value equals the intraperitoneal fluid volume after 2 hours minus the volume of fluid injected. Then, the collected peritoneal fluid samples were centrifuged at 800 × g for 5 min. Glucose levels in the supernatant were measured by a detection kit (Beyotime Biotechnology, China, S0201S). The formula to measure glucose transport function was previously described [3, 4]. We corrected the results for animal weight.

**Intraperitoneal delivery of liposomal siRNAs *in vivo*** Small interfering RNA (siRNA) can mediate targeted mRNA transcript cleavage, repress gene expression, and compromise gene function within living cells [6, 7]. siRNA drugs have been tested in animals by the local administration route and in phase I-III clinical trials of drugs. To improve siRNA efficacy *in vivo*, siRNAs for i.p. administration in mice were 2′-O-Me-modified by GenePharma (GenePharma Co., Ltd.). The transfection reagent LipoRNAi™, which was based on nanomaterials, was purchased from Beyotime (Beyotime Biotechnology Co., Shanghai, China, C0535) and used for the transfection of siRNA, miRNA, and other small nucleic acids in animals and gene therapy. Three complementary oligodeoxynucleotides of siRNA targeted at mouse H19 were designed (siH19-1#5′-UGAAGGGGCGAGGAUGACAGGU-3′; siH19-2#5′-CAUUCAUCCCGGUUACUUUTT-3′; siH19-3#5′-CCACCGUAAUUCACUUAGATT-3′), along with a negative siRNA (5′-UUCUCCGAACGUGUCACGUTT-3′). First, we verified the H19-targeting efficiency of the siRNA. The nanomaterial-encapsulated modified siRNAs (2 mg/kg) were injected into the peritoneal cavities of mice and retained by intraperitoneal indwelling for 24 h (*n=6* mice). Then, the peritoneal cells were extracted by EDTA-trypsin digestion. Total RNA was extracted by the phenol/chloroform method, followed by qRT‒PCR. We chose siH19-3 for the following experiment. Mice were randomly divided into three groups (*n=6 mice*): the siNC group, the PD + siNC group, and the PD + siH19-3 group.

**Cell culture** Human peritoneal mesothelial cells, the MeT-5A cell line, were purchased from ATCC. MET-5A cells were grown in M199 medium (BasalMedia Co., L640KJ) supplemented with 10% foetal bovine serum, 1% penicillin, and streptomycin. Mouse primary peritoneal mesothelial cells were obtained by 0.25% trypsin-EDTA (Epizyme Co., China, CB011) digestion of the parietal peritoneum, sorted by flow cytometry [8, 9], and then cultured in DMEM supplemented with 20% foetal bovine serum as previously described. Cells were incubated at 95% relative humidity and 5% CO2 at 37 °C. The dialysate applied in the clinic contains 1.5%, 2.5%, or 4.25% glucose. Cells were stimulated with 1.5%, 2.5%, or 4.25% D-glucose for 72 h and treated with tamoxifen citrate [3, 10] (5 μM, MedChemExpress).

**Peritoneal histology and** **immunofluorescence** Peritoneum samples were fixed in 4% paraformaldehyde for histologic analysis at 4 °C for 24 h and embedded in paraffin. Sections (4 μm thick) were sliced and stained with the Masson trichrome technique. After dewaxing, antigen retrieval, and permeabilization, paraffin-embedded sections were incubated with anti-ESR1 antibody (Santa Cruz Biotechnology, Inc., sc-8005) overnight and secondary antibody for 1 h in the dark. Finally, an anti-fluorescence quenching blocking solution (Beyotime Biotechnology, Shanghai, China) containing DAPI was added. Two independent investigators assessed the degree of fibrosis. The final score was the average value. Quantitative analysis was conducted using ImageJ software.

**Cell viability** Cell viability was detected by Cell Counting Kit-8 (CCK8, MedChemExpress, USA). Cells were seeded in a 96-well plate at 5×10^3^ cells/well and incubated overnight. Then, the medium was replaced by 100 μL of fresh medium containing 4.25% HG, with or without TAM (5 μM), followed by incubation for 24, 48, and 72 hours. Cells transiently transfected with siRNAs were digested with EDTA-Trypsin and seeded into 96-well plates after successful transfection to detect cell viability. Ten microlitres of CCK8 reagent was added to each well and incubated at 37 °C for 2 hours. The optical density (OD) of each well was measured at 450 nm by a multimode reader (BioTek). Three independent experiments were conducted.

**Transfection of siRNAs in MeT-5A cells** For the knockdown of H19 expression in Met-5A cells, three complementary oligodeoxynucleotides of siRNA and a negative siRNA were designed and purchased from IBS company (Shanghai Integrated Biotech Solutions Co. Ltd). The interfering sequence with the highest inhibition efficiency was chosen in the subsequent experiments. siRNAs targeting VEGFA in the MeT-5A cell line were also purchased. According to the manufacturer’s instructions, Lipofectamine 3000 reagent (Invitrogen, USA) was used to carry out transient transfection of siRNAs in Met-5A cells, which were incubated for 48 h before use in experiments. RT‒qPCR was performed to measure transfection efficiency.( siH19-1#5′-CCAACAUCAAAGACACCAUTT-3, siH19-2#5′CCUCUAGCUUGGAAAUGAATT3′, siH19-3#5′-UAAGUCAUUUGCACUGGUUTT-3′, siVEGFA-1#5′GGAGUACCCUGAUGAUGGAUCUU3′, siVEGFA-2#5′-ACCUCACCAAGGCCAGCACUU-3′, siVEGFA-3#5′-GGCGAGGCAGCUUGAGUUAAA-3′, siESR1-1#5′- CGAGUAUGAUCCUACCAGACC -3′, siESR1-2#5′-GCUACUGUGCAGUGUGCAAUG -3′, si-p300-1#5′-GGACUACCCUAUCAAGUAAAU-3′, si-p300-2#5′-CGACUUACCAGAUGAAUUAAU -3′, negative siRNA 5′-UUCUCCGAACGUGUCACGUTT-3)

**Construction of lentivirus-infected stable cell lines** Lentiviruses overexpressing H19 or NC (LV5/EF-1a/GFP/Puro) were purchased from GenePharma. MeT-5A cells were infected with LV-H19 or LV-NC and selected with puromycin (2 μg/ml) for 72 h. qRT‒PCR was conducted to assess the overexpression efficiency.

**RNA extraction and quantitative real-time PCR (qPCR)** Total RNA was extracted from cells and peritoneal tissues by TRIzol (Invitrogen, Carlsbad, CA). The concentration and purity of RNA were determined by the 260/280 nm absorbance. RNA was reverse transcribed with a cDNA Synthesis Kit (Vazyme Biotech Co., Ltd., Nanjing, China). SYBR Green PCR kits (Yeasen, China) were used for real-time qPCR. The primers are listed in Table 2. Actin RNA was used as control by 2^-ΔΔCt method.

**Western blot analysis** Lysates from cells and peritoneum were extracted with RIPA Lysis Buffer (Epizyme Biotech Co., Ltd.) and centrifuged at 13,000 × g for 5 min. The protein concentration was measured using a bicinchoninic acid (BCA) assay kit (Beyotime Biotechnology, China). Equal amounts of protein were loaded into 4-12% PAGE gels (Genscript Biotech, China) and transferred onto a nitrocellulose membrane (NC; GE, GER). Membranes were blocked in 5% nonfat milk at room temperature for 2 hours and then incubated at 4 °C overnight with primary antibodies specific for ESR1 (Santa Cruz Biotechnology, Inc., sc-8005), ESR2 (Boster, A00786-1), α-SMA (Abcam, ab7817), Col2A1 (Abclonal, A1560), VEGFA (Proteintech, 19003-1-AP), E-cad (Proteintech, 20874-1-AP), Vimentin (Boster, PB9359), PI3K (Proteintech, 27921-1-AP), p-AKT (Cell Signaling Technology, 4060S), p-p38 (Abways, CY6391), p-smad2 (Cell Signaling Technology, 18338S), and p-smad3 (Cell Signaling Technology, 9520S). After three washes with TBST, the membranes were incubated with IRDye 700/800‐conjugated secondary antibodies. The results were visualized and analysed by an Odyssey Fluorescence Imaging System (Gene, USA).

**Separation of nuclear and cytoplasmic proteins** Nucleus and cytoplasmic extraction kit was abtained from Thermo Fisher (78833). After the separation of nuclear and cytoplasmic proteins, we verified that there was no Actin protein in the nucleus and no Histone H3 protein in the cytoplasm.

**ELISA** The VEGFA protein in the cell supernatant was detected by the Human and Mouse VEGF ELISA Kit (Shangahi absin biotechnology Co., abs510008, abs520008). Specific anti-human VEGF antibody was precoated on a high-affinity ELISA plate. VEGF in samples binds to the solid-phase antibody and the detection antibody to form an immune complex. After adding streptavidin-HRP, substrate, and stop solution, the absorbance value was measured at 450 nm (reference calibration wavelength 540 nm or 570 nm).

**Detection of cell proliferation by EdU** EdU (5-ethynyl-2'-deoxyuridine) is a thymidine analogue that can replace thymidine (T) incorporation during cell proliferation. This specific reaction can quickly and accurately detect the DNA replication activity of cells. We performed the experiment following the instructions of the EdU Apollo Kit (Guangzhou Ribo Biotechnology Co., Ltd.). After two hours of incubation with EdU, the cells were fixed with 4% paraformaldehyde and stained with Apollo and DAPI. Finally, image capture was performed with a fluorescence microscope.

**Detection of cell apoptosis by TUNEL** A TUNEL Apoptosis Detection Kit (Vazyme, Nanjing, China) was used to evaluate apoptosis according to the manufacturer’s instructions. Cells were fixed in 4% paraformaldehyde, permeabilized with 0.3% Triton X-100 solution, and incubated with a mixed solution of TdT buffer. DAPI was used to stain nuclei. Finally, the cells were observed and photographed under an inverted fluorescence microscope.

**Chromatin immunoprecipitation (ChIP)** A chromatin immunoprecipitation (ChIP) assay was used to study the binding of proteins to gene promoter sites. We performed ChIP (Millipore) according to the manufacturer’s instructions. In brief, cross-linked chromatin was sonicated into 200-1000 bp fragments. Chromatin was immunoprecipitated with anti-ESR1 (Santa Cruz, sc-8002), anti-H3K27ac (Abcam, ab4729), and p300 (Abcam, ab275378) antibodies. Normal mouse immunoglobulin G (IgG) was used as a negative control. qRT‒PCR was conducted using SYBR Green PCR kits (Yeasen, China). The *JASPAR* database was used to predict the binding site to the promoter region. The relative protein-binding enrichment was relative to the input, and expression fold changes were calculated using 2^-ΔCt method. The gene-specific primers with the sequences are listed in Table 3.

**Dual‐luciferase reporter assay** pSI-h-LncH19-3UTR-mut and pSI-h-LncH19-3UTR-wt plasmids carrying the firefly and Renilla luciferase genes were constructed by Hanbio Biotechnology Co. Ltd. (Shanghai, China). Plasmids were transfected using Lipofectamine 3000. After successful transfection, cells were treated with different stimuli. Firefly luciferase and Renilla luciferase activities were tested separately in the same sample to measure promoter activation. OD values were detected using the Dual-luciferase Reporter Assay System (Promega, Madison, USA) in a microplate reader.

**Cytoplasmic and nuclear RNA extraction** A Cytoplasmic and Nuclear RNA Purification Kit (Norgen Bioteck, Canada) was used to determine the localization of H19. All procedures were performed in an RNase-free environment. Lysis buffer J (containing β-mercaptoethanol) was added to separate cytoplasmic and nuclear components. After high-speed centrifugation, the supernatant contained cytoplasmic RNA, and the nuclear RNA fraction was in the pellet. Then, quantification was performed by qRT‒PCR. After the separation of nuclear and cytoplasmic RNA, the CT value of U6 RNA in the cytoplasm was >40 cycles, and the CT value of Actin in the nucleus was >40 cycles.

**RNA immunoprecipitation (RIP)** To investigate protein‒RNA binding, we performed RIP experiments using the Magna RIP RNA-Binding Immunoprecipitation Kit (Millipore, Bedford, MA, USA) according to the manufacturer’s instructions. The antibody used for RIP was p300 (Abcam, ab275378). Normal mouse immunoglobulin G (IgG) and actin RNA were used as negative controls. First, the cell lysate was obtained with RIP Lysate Buffer. Then, magnetic bead antibodies were prepared to immunoprecipitate RNA-binding proteins. RNA was extracted and purified, and RNA enrichment was determined by qRT‒PCR and normalized to the input control.

**RNA FISH** We performed fluorescence in situ hybridization (FISH) to test the localization of H19 in MeT-5A cells. Biotin-labelled probes targeting human H19 (5’ to 3’: 1#CCGAGAAGATGTCACCTTTGCTAAC, 2#CCTGCCAGACTCCAGATG, 3# TCCTGTAACCAAAAGTGACCGGG) were synthesized by Genepharma Co., and the RNA FISH Kit SA-Biotin System was used to detect the signal (GenePharma Co., China). Cy3 conjugated to streptavidin protein was bound to biotin-labelled probes *in vitro* at 37 °C for 30 min. The oligonucleotide probes and the nucleic acid sequence in cells formed a complex based on the principle of base complementarity at 37 °C for 16 h, and the target signal was captured by a fluorescence microscope.

**RNA pull-down assay** RNA pull-down was conducted using the Pierce Magnetic RNA‒Protein Pull-Down Kit (Thermo Fisher Scientific). pcDNA3.1-H19 and pcDNA3.1-vector were used as templates for performing polymerase chain reactions using Phanta Flash Master Mix (Vazyme, Nanjing, China). PCR products were subjected to agarose gel electrophoresis and purified by a DNA Extraction Mini Kit (Vazyme, Nanjing, China). For *in vitro* transcription, the T7 High Yield RNA Transcription Kit was used (Vazyme, Nanjing, China). Then, the single biotinylated nucleotide was attached to the 3´ terminus of the RNA strand by a Pierce™ RNA 3' End Desthiobiotinylation Kit (Thermo Fisher Scientific). Procedures for the enrichment of labeled RNA-binding proteins were carried out according to the manual of the Pierce Magnetic RNA‒Protein Pull-Down Kit (Thermo Fisher Scientific).

**Statistical analysis** Data are expressed as means ± SD. Statistical analyses were performed with GraphPad Prism (version 8.0, GraphPad Software, San Diego, CA). The Kolmogorov-Smirnov normality test was performed to test value distribution. A two-tailed Student’s t-test was used for the comparison of the two groups. One-way ANOVA was used followed by Dunnett's T3. Tukey's multiple comparisons were tested following Two-way ANOVA. The correlation between the two variables was analyzed by Pearson correlation. Significance was defined as P < 0.05.

**Table 1a Patients’ information**

| CON | Name | Sex | Age |
| --- | --- | --- | --- |
| 1 | ZH | male | 45 |
| 2 | GFB | male | 56 |
| 3 | LR | female | 42 |
| 4 | XF | female | 54 |
| 5 | TXD | male | 68 |
| 6 | PLY | male | 73 |
| PD | Name | Sex | Age |
| 1 | ZYM | male | 48 |
| 2 | LX | male | 52 |
| 3 | XDJ | male | 64 |
| 4 | LBD | female | 64 |
| 5 | ZGF | female | 72 |
| 6 | LD | female | 56 |

**Table 1b Patients’ information**

|  |  | n | ESR1  RNA level (Mean ± SD) | *P* | H19  RNA level (Mean ± SD) | *P* |
| --- | --- | --- | --- | --- | --- | --- |
| Gender |  |  |  |  |  |  |
| Male |  | 25 | 0.804 ± 0.299 | 0.854 | 0.808 ± 0.306 | 0.570 |
| Female |  | 12 | 0.783 ± 0.3563 |  | 0.750 ± 0.243 |  |
| Age (years) | >=60y | 22 | 0.795 ± 0.260 | 0.966 | 0.790 ±0.245 | 0.965 |
|  | <60y | 15 | 0.800 ± 0.389 |  | 0.787 ± 0.346 |  |
| PD duration (months) | >=60 | 21 | 0.981 ± 0.236 | <0.001 | 0.881 ± 0.234 | 0.0229 |
|  | <60 | 16 | 0.556 ± 0.231 |  | 0.667 ± 0.305 |  |
| Peritoneal Equilibrium  Test | L&LA (D/P Cr <0.65 )  H&HA (D/P Cr >=0.65 ) | 19  18 | 0.626 ± 0.249  0.978 ± 0.276 | <0.001 | 0.605 ± 0.237  0.983 ± 0.189 | <0.001 |

Abbreviations: D/P Cr: dialysate/plasma ratio of creatinine.

**Table 2 Primers for qRT-PCR**

| mRNA | Primer Pairs |
| --- | --- |
| Human Actin | Forward: CTCCATCCTGGCCTCGCTGT  Reverse: GCTGTCACCTTCACCGTTCC |
| Human H19 | Forward: CTCCCTCTTCTTCTTTTTCATC  Reverse: CGCACACTCGTACTGAGACT |
| Human VEGFA | Forward: AGGGCAGAATCATCACGAAGT  Reverse: AGGGTCTCGATTGGATGGCA |
| Human ESR1 | Forward: TACTGCATCAGATCCAAGGGAA  Reverse: CCTCGGGGTAGTTGTACAC |
| Human ESR2 | Forward: GCCGACAAGGAGTTGGTACA  Reverse: ACTTGGTCGAACAGGCTGAG |
| Mouse Actin | Forward: AACAGTCCGCCTAGAAGCAC  Reverse: CGTTGACATCCGTAAAGACC |
| Mouse H19 | Forward: CGCTCCACTGACCTTCTAAAC  Reverse: GACGATGTCTCCTTTGCTAACT |
| Mouse ESR1 | Forward: CCCGCCTTCTACAGGTCTAAT  Reverse: CTTTCTCGTTACTGCTGGACAG |
| Mouse VEGFA | Forward: GCACATAGAGAGAATGAGCTTCC  Reverse: CTCCGCTCTGAACAAGGCT |
| Mouse ESR2 | Forward: CTGAGCCACCCAATGTGCTA  Reverse: CCTCATCCCTGTCCAGAACG |

**Table 3 Primers used in chromatin immunoprecipitation assay.**

| Promoters | Primer Pairs |
| --- | --- |
| Human VEGFA [11] | Forward: TCTTTAGCCAGAGCCGGGGT  Reverse: GGACACACAGATCTGTTGGA |
| Human H19-1 | Forward-1: CCACCCCTACTCTCCAGGAA  Reverse-1: CACACGTCTCTCTCACCCAG |
| Human H19-2 | Forward-2: GTCTGGGAGGGAGAAGTCCT  Reverse-2: ACAGTTCCAGCACACGTCTC |
| Mouse VEGFA | Forward: CATTTCGCGGTAGTGGCCTA  Reverse: CTTCTCACCGGTAACAGCGG |
| Mouse H19 | Forward: TACTGTGCCTCCGGTTGACT  Reverse: TGGGTGAGTCGTCACGTTTG |


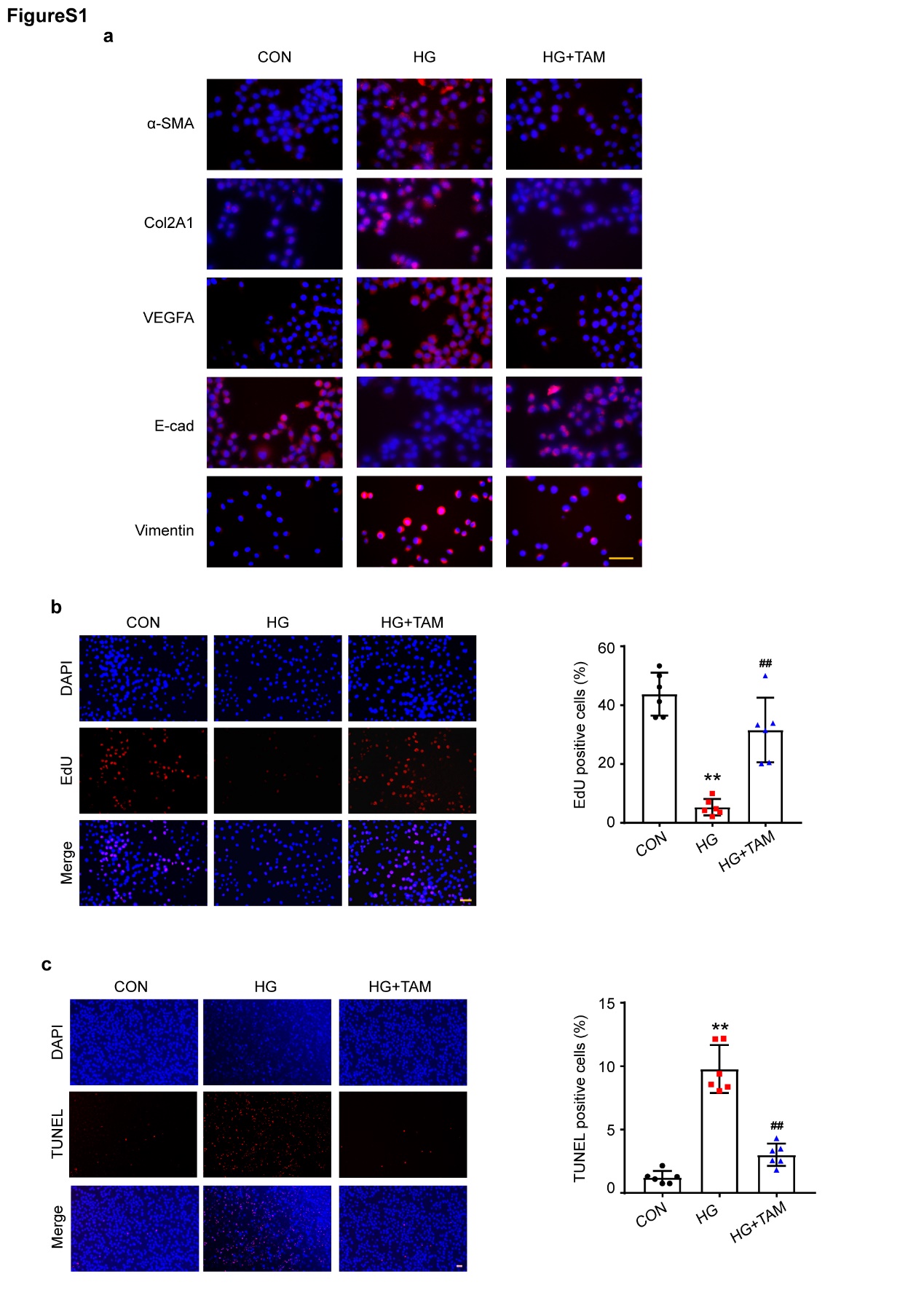


**Figure. S1** **Immunofluorescence merge images, EdU, and TUNEL staining of murine peritoneal primary cells. a** Merge pictures of immunofluorescence showing MMT markers in murine primary peritoneal cells. Scale bars = 100 μm. **b** EdU assay for the proliferation of mouse peritoneal primary cells and quantification of EdU-positive cells. Scale bars = 100 μm. n = 6. ***P* <0.01 vs. CON group, **^##^***P* <0.01 vs. HG group. **c** TUNEL Bright Red test showing apoptosis and quantification of TUNEL-positive cells. Scale bars = 100 μm. n = 6. ***P* <0.01 vs. CON group, **^##^***P* <0.01 vs. HG group. Values are the mean±SD. Pairwise comparison between groups was tested by Dunnett's T3 following One-way ANOVA.


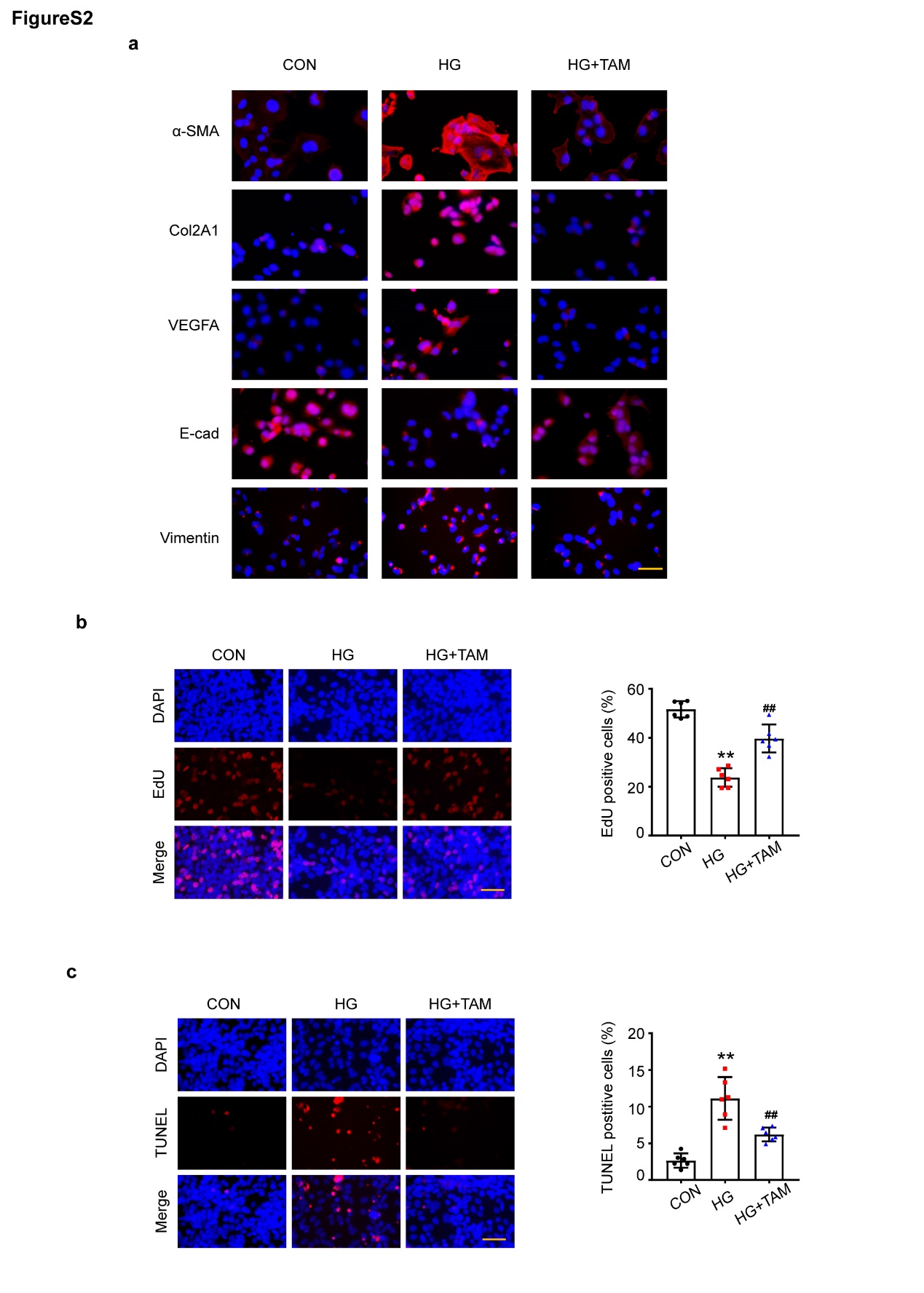


**Figure. S2** **Immunofluorescence merge images, EdU, and TUNEL staining of MeT-5A cells. a** Merge pictures of immunofluorescence indicating EMT markers in MeT-5A cells. Scale bars = 100 μm. **b** EdU assay for the proliferation of MeT-5A cells and quantification of EdU-positive cells by Image. Scale bars = 100 μm. n = 6. ***P* <0.01 vs. CON group, **^##^***P* <0.01 vs. HG group.**c** TUNEL Bright Red test showing apoptosis and quantification of TUNEL-positive cells. Scale bars = 100 μm. n = 6. ***P* < 0.01 vs. CON group, **^##^***P* <0.01 vs. HG group. Pairwise comparison between groups was tested by Dunnett's T3 following One-way ANOVA.


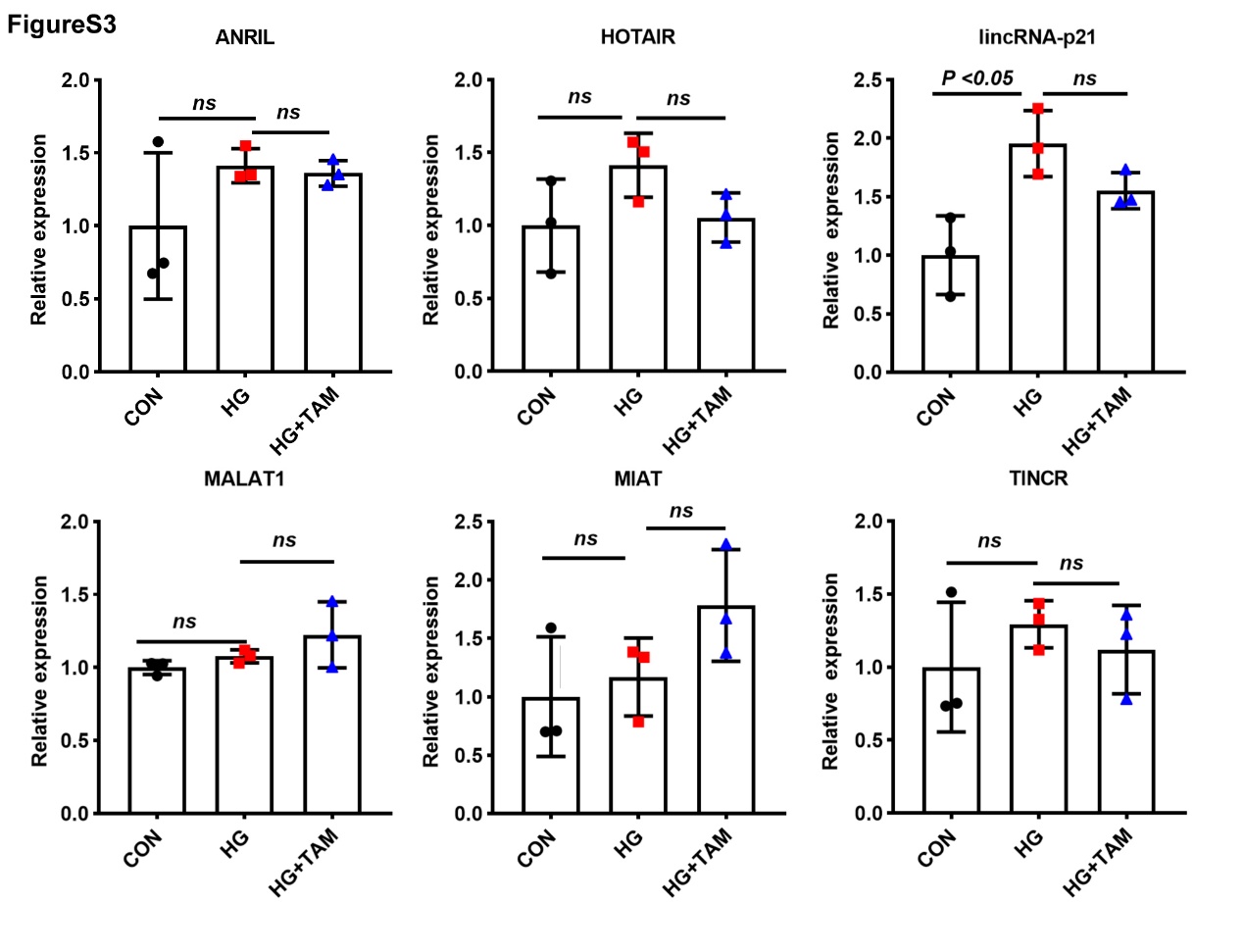


**Figure. S3 Fibrotic disease-related LncRNA expression changes in MeT-5A cells under HG stimulation and TAM treatment.**

The above experiments were repeated three times. Pairwise comparison between groups was tested by Dunnett's T3 following One-way ANOVA.


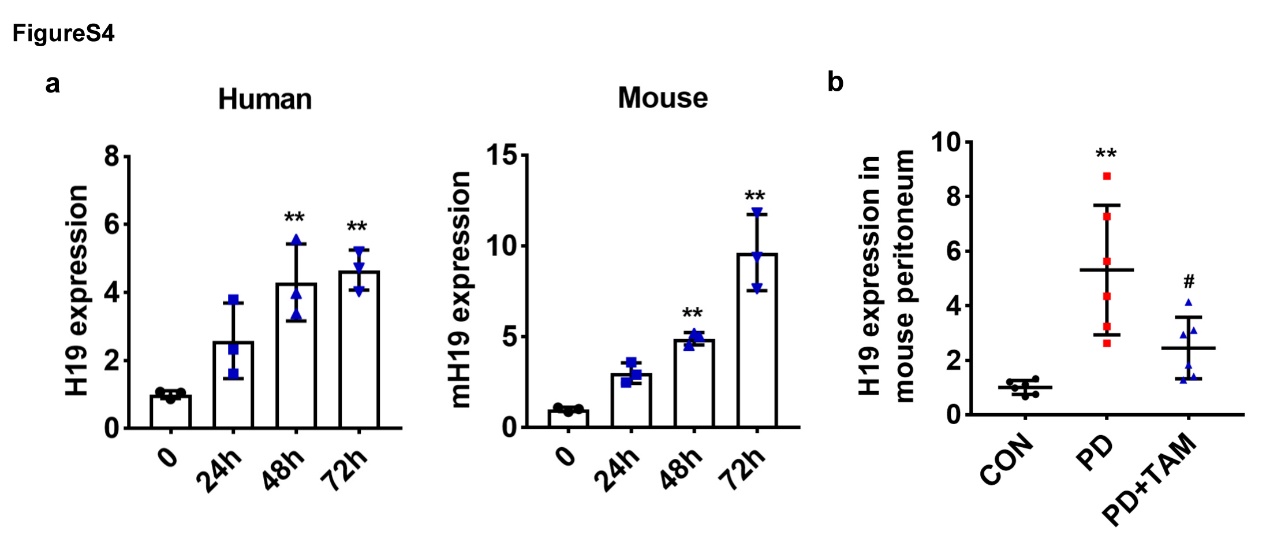


**Figure. S4 H19 expression changes a H19 expression changes by 4.25% glucose stimulation with time gradually extending.** n = 3. ***P* <0.01 vs. 0 h group. **b** H19 RNA level in tamoxifen administration model. ***P* <0.01 vs. CON group, **^#^***P* <0.05 vs. PD group. Pairwise comparison between groups was tested by Dunnett's T3 following One-way ANOVA.


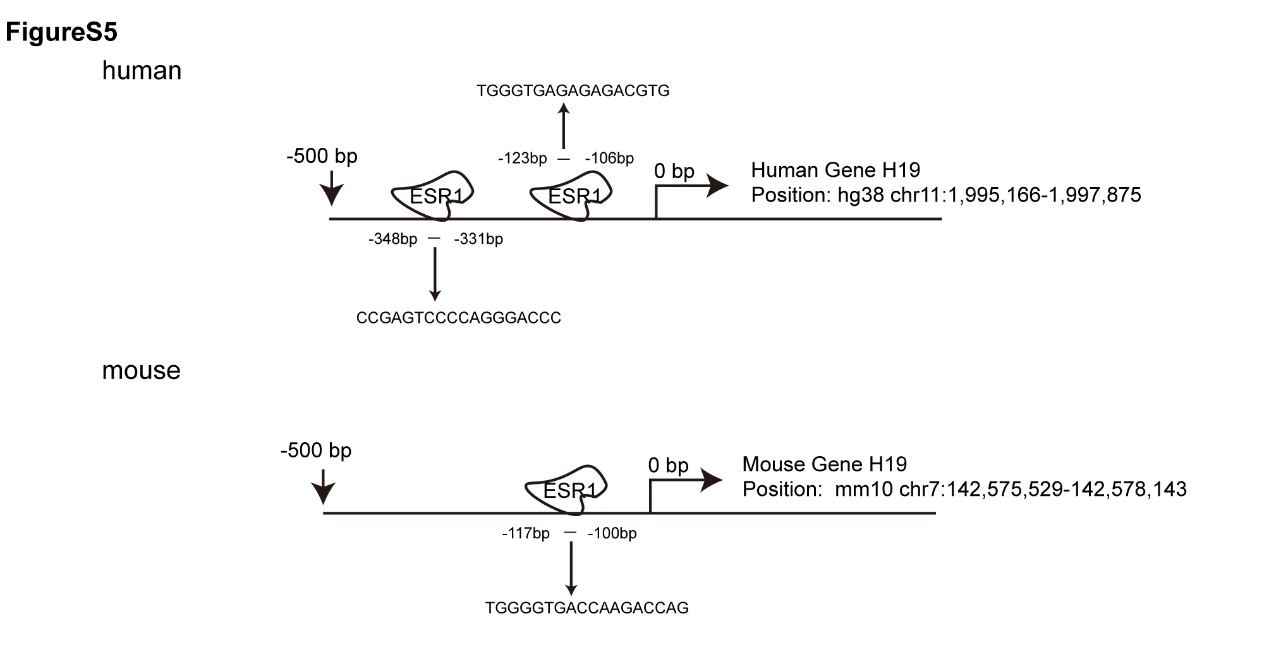


**Figure. S5 ESR1 binding site of *LncH19* promoter region by *JASPAR* and UCSC databases.**


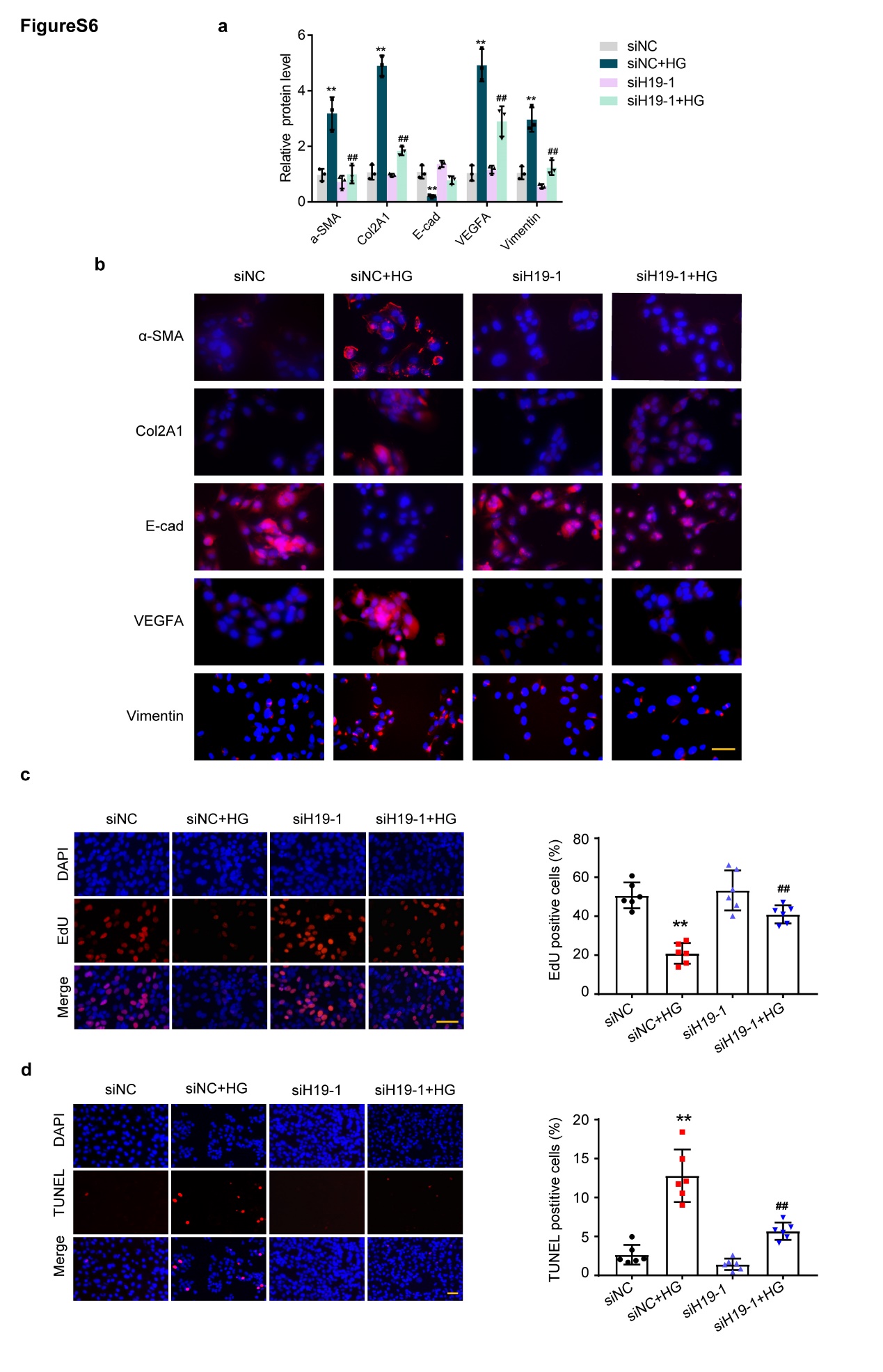


**Figure. S6** **Effects of siH19 against HG stimulated-MMT of MeT-5A cells** a Statistical analysis of Western Blot images. n=3. ***P* <0.01 vs. siNC group, **^##^***P* <0.01 vs. siNC+HG group. **b** Immunofluorescence merge images of EMT marker proteins. Scale bars = 100 μm. **c** EdU showing MeT-5A cell proliferation and percentages of positive cells. Scale bars = 100 μm. n=6. ***P* <0.01 vs. siNC group, **^##^***P* <0.01 vs. siNC+HG group. **d** TUNEL staining of MeT-5A apoptosis and statistic data analysis. Scale bars = 100 μm. n=6. ***P* <0.01 vs. siNC group, **^##^***P* <0.01 vs. siNC+HG group. Pairwise comparison between groups was tested by Dunnett's T3 following One-way ANOVA.


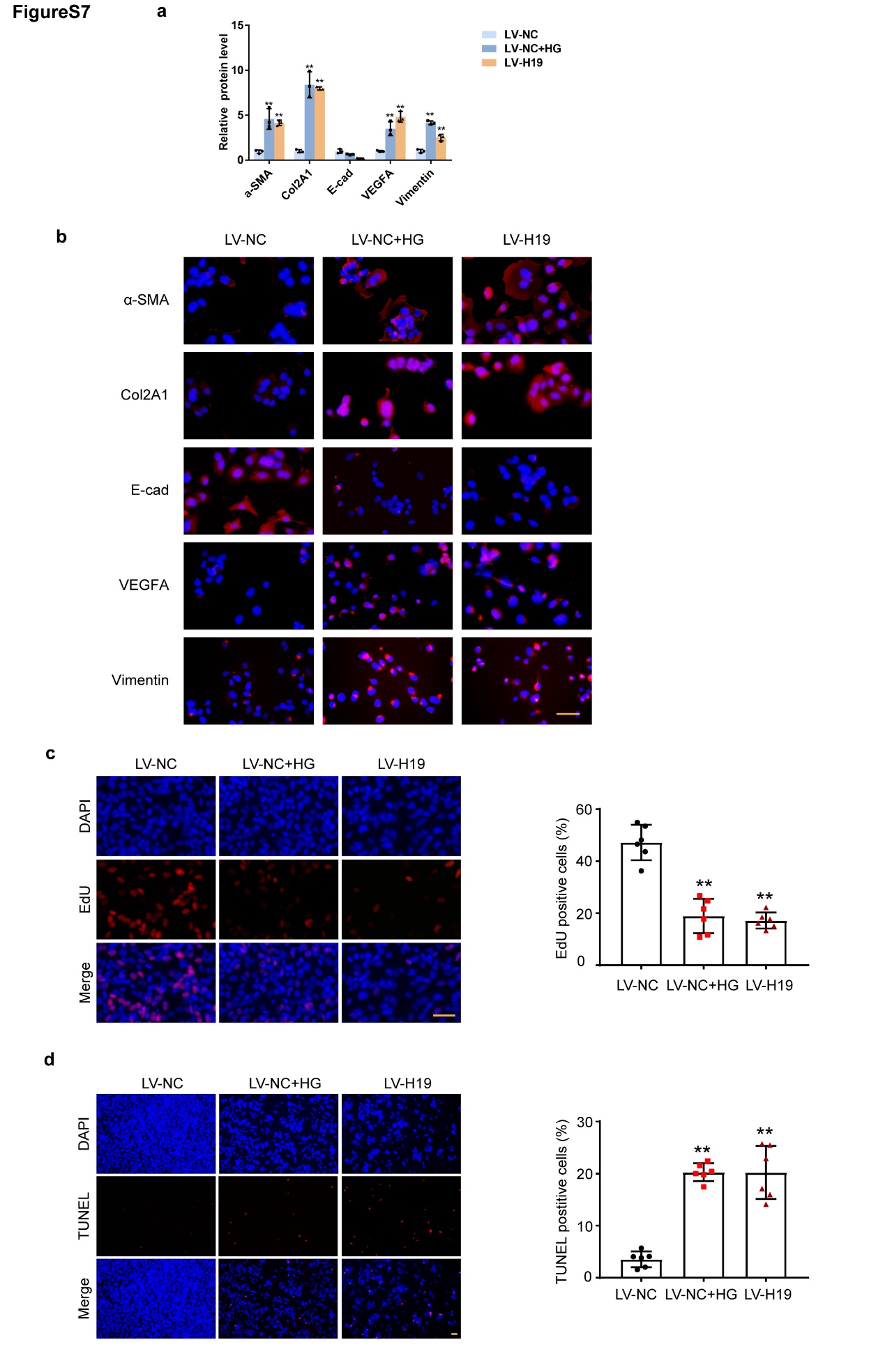


**Figure. S7** **Overexpression H19 promoted MMT of MeT-5A cells. a** Statistical analysis of Western Blot images from three independent experiments. n=3. ***P* <0.01 vs. LV-NC group. **b** Immunofluorescence merge images of EMT marker proteins. Scale bars = 100 μm. **c** EdU assay for the proliferation of MeT-5A cells and quantification of EdU-positive cells by ImageJ software. Scale bars = 100 μm. n=6. ***P* <0.01 vs. LV-NC group. **d** TUNEL Bright Red test showing apoptosis and quantification of TUNEL-positive MeT-5A cells by ImageJ software. n=6. ***P* <0.01 vs. LV-NC group. Pairwise comparison between groups was tested by Dunnett's T3 following One-way ANOVA.


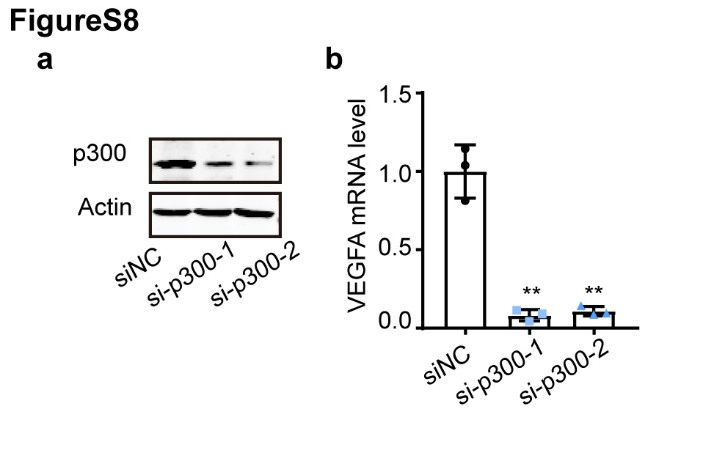


**Figure. S8 Reducing p300 in MeT-5A cells suppressed VEGFA mRNA level. a** Western Blot showing the knockdown efficacy of siRNAs targeting p300 in MeT-5A cells. **b** VEGFA mRNA levels detected by qRT-PCR. n =3. ***P* <0.01 vs. siNC group. Pairwise comparison between groups was tested by Dunnett's T3 following One-way ANOVA.


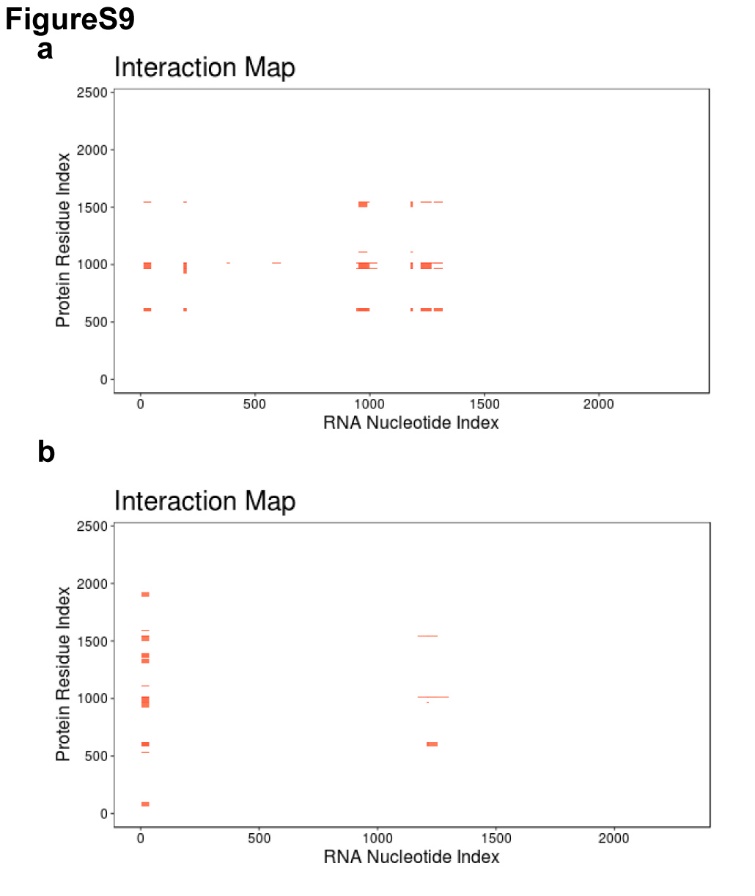


**Figure. S9 Predictions of H19 and p300 binding in human and mice.**


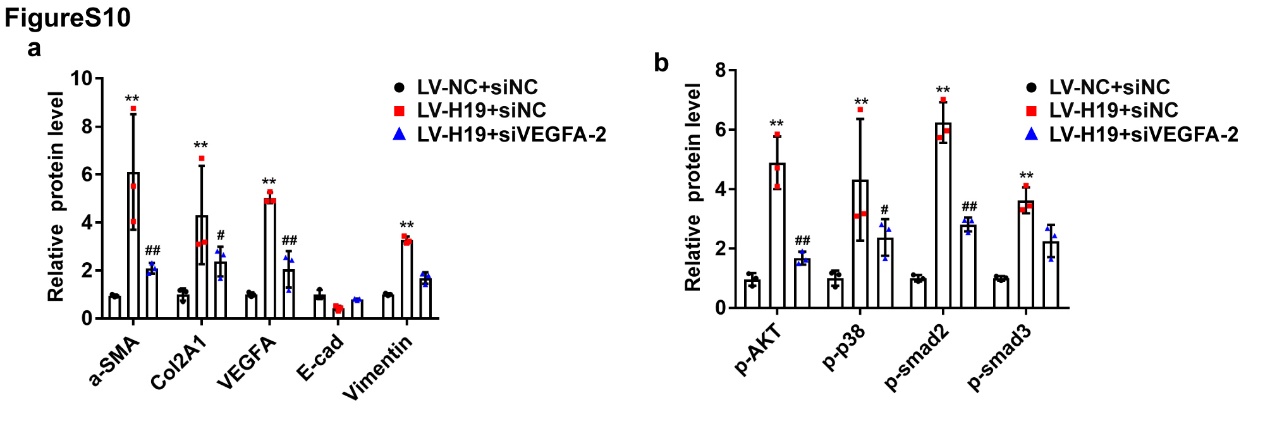


**Figure. S10 Statistical analysis of MMT protein changes in LV-H19 infected MeT-5A cells. a** Western blot statistical analysis of fibrosis-related protein changes. **b** H19-induced activation pathways. n =3 ***P* <0.01 vs. LV-NC+siNC group, **^#^***P* <0.05, **^##^***P* <0.01 vs. LV-H19+siNC group.


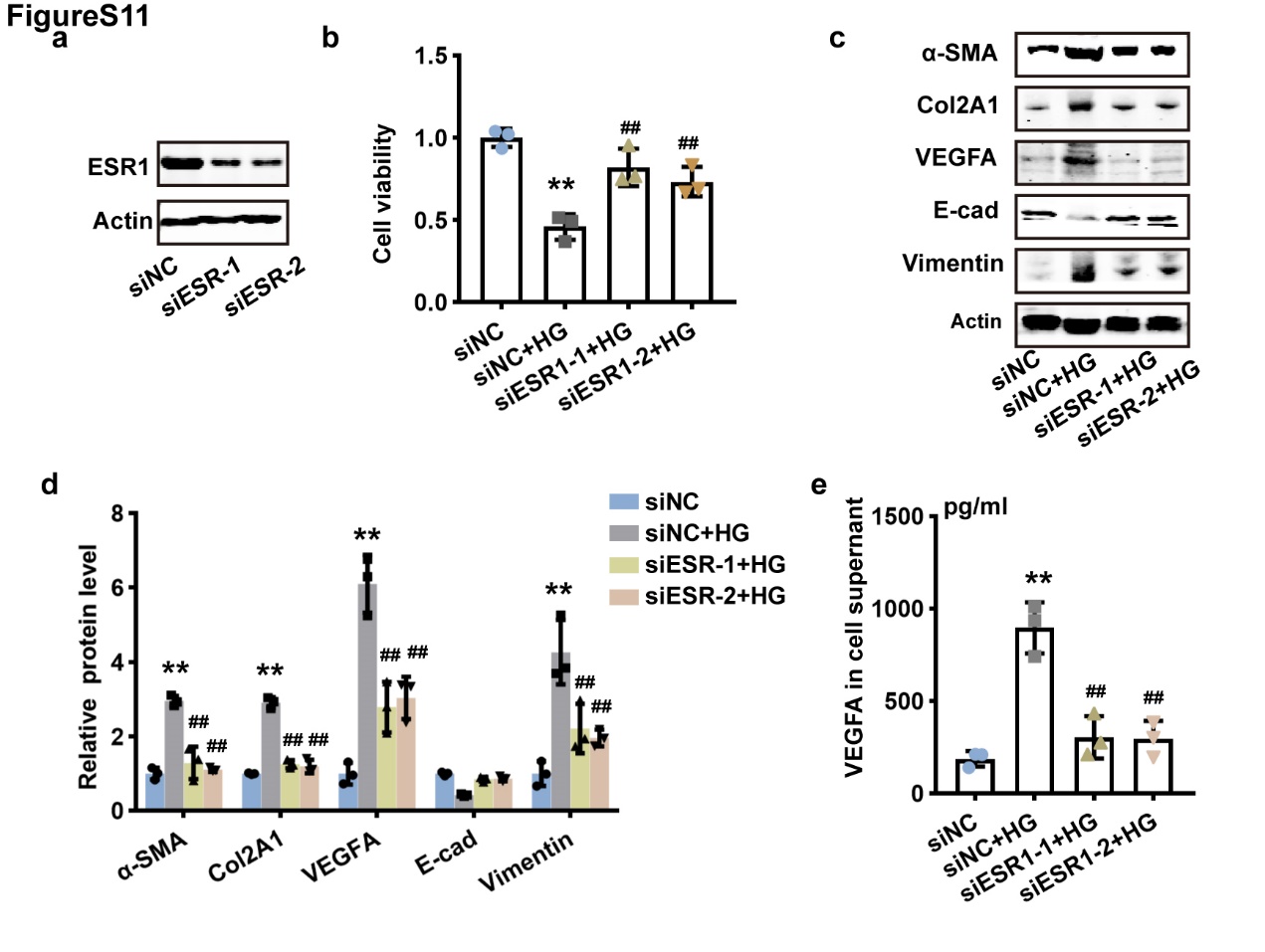


**Figure. S11 HG-induced pro-fibrotic effect was reversed by siESR1 in MeT-5A. a** Western Blot showing the knockdown efficacy of siRNAs targeting ESR1 in MeT-5A cells. **b** CCK-8 showing cell viability. **c** and **d** Western blot of EMT marker proteins and statistical analysis. **e.** ELISA assay detecting VEGFA release from MeT-5A cells. n =3. ***P* <0.01 vs. siNC group, **^##^***P* <0.01 vs. siNC+HG group. Pairwise comparison between groups was tested by Dunnett's T3 following One-way ANOVA.


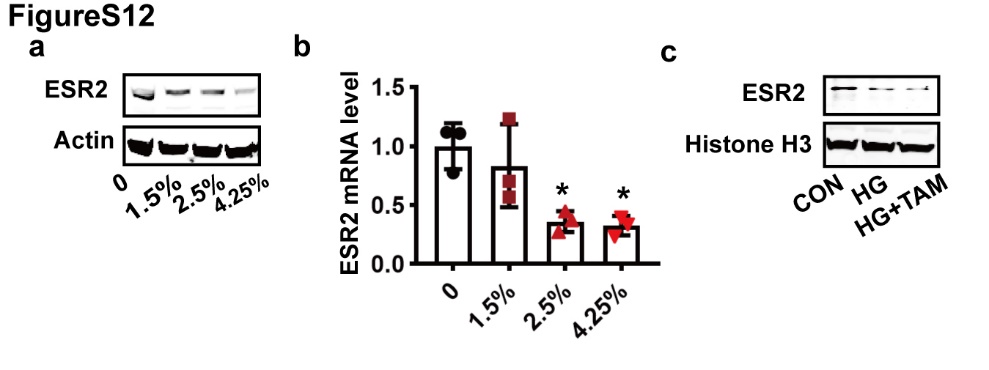


**Figure. S12** **ESR2 total protein and mRNA levels were decreased in a glucose-dependent manner, while tamoxifen did not affect its nuclear expression in MeT-5A cells. a** ESR2 total protein expression in MeT-5A cells. **b** mRNA level in MeT-5A cells stimulated with 0,1.5%,2.5%, and 4.25% glucose. n =3. **P* <0.01 vs. 0 h group. **c** Western Blot analyzed ESR2 in the MeT-5A nucleus. n =3. ***P* <0.01 vs. siNC group, **^##^***P* <0.01 vs. siNC+HG group. Pairwise comparison between groups was tested by Dunnett's T3 following One-way ANOVA.


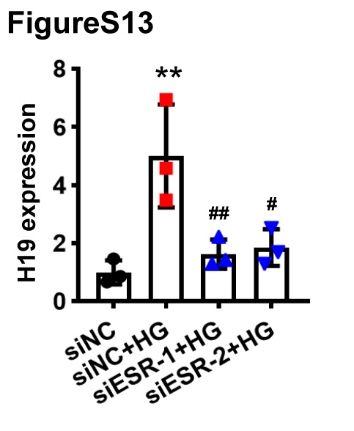


**Figure. S13 H19 RNA level was decreased by siRNA targeting ESR1 in MeT-5A cells****.**

***P* <0.01 vs. siNC group, **^##^***P* <0.01 vs. siNC+HG group, **^#^***P* <0.01 vs. siNC+HG group. Pairwise comparison between groups was tested by Dunnett's T3 following One-way ANOVA.


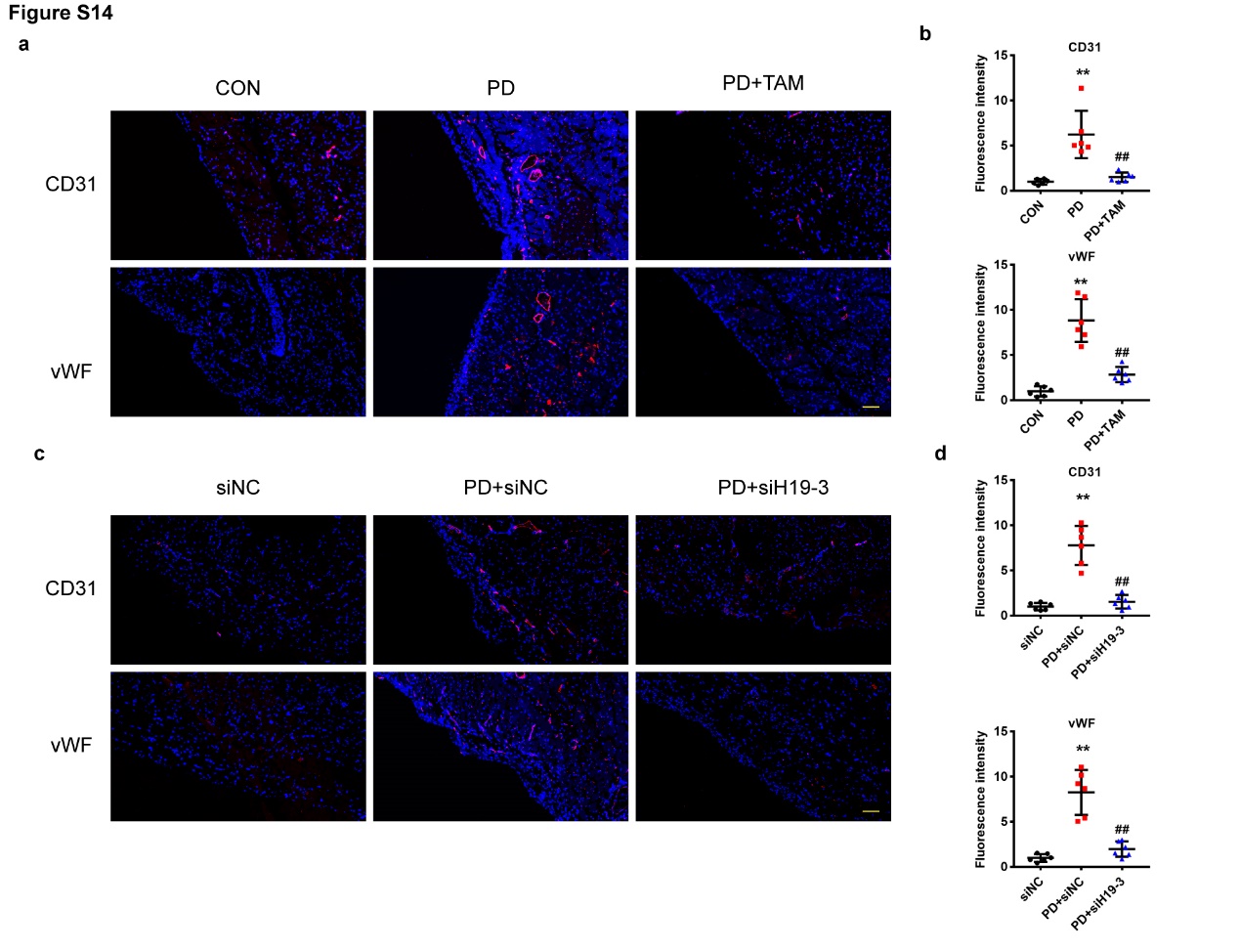


**Figure S14 Angiogenesis markers in mouse models. a** and **c.** CD31 and vWF (markers of angiogenesis) in mouse peritoneum. Scale bars = 100 μm. **b.** Relative quantification of fluorescence intensity. ***P* <0.01 vs. CON group, **^##^***P* <0.01 vs. PD group. **d.** Relative quantification of fluorescence intensity. ***P* <0.01 vs. siNC group, **^##^***P* <0.01 vs. PD+siNC group. Pairwise comparison between groups was tested by Dunnett's T3 following One-way ANOVA.


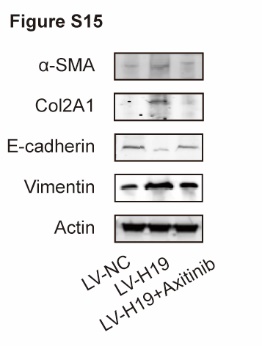


**Figure S15 Western Blot analysis of MeT-5A cells treated with Axitinib.**

**References**

1. Chen YT, Chang YT, Pan SY, Chou YH, Chang FC, Yeh PY, et al. Lineage tracing reveals distinctive fates for mesothelial cells and submesothelial fibroblasts during peritoneal injury. *J Am Soc Nephrol.* 2014;25(12):2847-58. doi: 10.1681/ASN.2013101079.

2. Kitamura M, Nishino T, Obata Y, Furusu A, Hishikawa Y, Koji T, et al. Epigallocatechin gallate suppresses peritoneal fibrosis in mice. *Chem Biol Interact.* 2012;195(1):95-104. doi: 10.1016/j.cbi.2011.11.002.

3. Silva F, Costalonga EC, Silva C, Carreira A, Gomes SA, Sogayar MC, et al. Tamoxifen and bone morphogenic protein-7 modulate fibrosis and inflammation in the peritoneal fibrosis model developed in uremic rats. *Mol Med.* 2019;25(1):41. doi: 10.1186/s10020-019-0110-5.

4. Li L, Shen N, Wang N, Wang W, Tang Q, Du X, et al. Inhibiting core fucosylation attenuates glucose-induced peritoneal fibrosis in rats. *Kidney Int.* 2018;93(6):1384-96. doi: 10.1016/j.kint.2017.12.023.

5. Lu H, Chen W, Liu W, Si Y, Zhao T, Lai X, et al. Molecular hydrogen regulates PTEN-AKT-mTOR signaling via ROS to alleviate peritoneal dialysis-related peritoneal fibrosis. *FASEB J.* 2020;34(3):4134-46. doi: 10.1096/fj.201901981R.

6. Howard KA, Paludan SR, Behlke MA, Besenbacher F, Deleuran B, Kjems J. Chitosan/siRNA nanoparticle-mediated TNF-alpha knockdown in peritoneal macrophages for anti-inflammatory treatment in a murine arthritis model. *Mol Ther.* 2009;17(1):162-8. doi: 10.1038/mt.2008.220.

7. Huang Y, Zheng S, Guo Z, de Mollerat du Jeu X, Liang XJ, Yang Z, et al. Ionizable liposomal siRNA therapeutics enables potent and persistent treatment of Hepatitis B. *Signal Transduct Target Ther.* 2022;7(1):38. doi: 10.1038/s41392-021-00859-y.

8. Busnadiego O, Loureiro-Álvarez J, Sandoval P, Lagares D, Dotor J, Pérez-Lozano ML, et al. A pathogenetic role for endothelin-1 in peritoneal dialysis-associated fibrosis. *J Am Soc Nephrol.* 2015;26(1):173-82. doi: 10.1681/ASN.2013070799.

9. Strippoli R, Loureiro J, Moreno V, Benedicto I, Pérez Lozano ML, Barreiro O, et al. Caveolin-1 deficiency induces a MEK-ERK1/2-Snail-1-dependent epithelial-mesenchymal transition and fibrosis during peritoneal dialysis. *EMBO Mol Med.* 2015;7(1):102-23. doi: 10.15252/emmm.201404127.

10. Loureiro J, Sandoval P, del Peso G, Gónzalez-Mateo G, Fernández-Millara V, Santamaria B, et al. Tamoxifen ameliorates peritoneal membrane damage by blocking mesothelial to mesenchymal transition in peritoneal dialysis. *PLoS One.* 2013;8(4):e61165. doi: 10.1371/journal.pone.0061165.

11. Cheng YC, Chiang HY, Cheng SJ, Chang HW, Li YJ, Shieh SY. Loss of the tumor suppressor BTG3 drives a pro-angiogenic tumor microenvironment through HIF-1 activation. *Cell Death Dis.* 2020;11(12):1046. doi: 10.1038/s41419-020-03248-5.
